# Light-activated, transcription-guided reconfiguration, aggregation, and intercommunication of structurally compartmentalized DNA condensates

**DOI:** 10.1039/d6sc06136b

**Published:** 2026-10-30

**Authors:** Shijun Xu, Yichen Han, Yunlong Qin, Zhenliang Tian, Yunxiao Wang, Itamar Willner, Fan Xia, Fujian Huang

**Affiliations:** a State Key Laboratory of Geomicrobiology and Environmental Changes, Faculty of Materials Science and Chemistry, China University of Geosciences Wuhan 430074 China xiafan@cug.edu.cn huangfj@cug.edu.cn; b The Institute of Chemistry, The Hebrew University of Jerusalem Jerusalem 91904 Israel itamar.willner@mail.huji.ac.il

## Abstract

A versatile concept for assembling photoresponsive microcompartmentalized phase-separated condensates as protocell models is introduced. The systems demonstrate the phototriggered activation of transcription machineries inducing dynamic switchable or transient spatiotemporal reconfiguration, aggregation/deaggregation, and intercommunication of microcompartmentalized condensates. The microcompartments are generated by competitive invasion of barcode-tethered cyanuric acid/polyadenine coacervates with *o*-nitrobenzyl phosphate ester caged DNA hairpin structures conjugated to strands exhibiting variable hybridization lengths to the barcode tethers. Photochemical uncaging of the hairpin structures results in the formation of constitutional compartments consisting of transcription templates or compartments composed of rearrangeable transcription template constituents. In the presence of T7 RNAp/NTPs, the transcription machineries in the respective compartments are activated, leading to RNA strands signaling dynamic reconfiguration of the microcompartments. By coupling RNase H to the T7 RNAp/NTP machineries, switchable or transient reconfiguration of the microcompartments is demonstrated. Moreover, by engineering a palindromic, self-complementary domain in one of the microcompartment constituents, cyclic switching or transient reconfiguration of the microcompartments, accompanied by reversible switchable/transient aggregation/deaggregation of the condensates, is demonstrated. Also, by employing two different microcompartmentalized condensates modified with complementary barcode tethers, aggregation of the condensates accompanied by the auxiliary transcription machinery-induced RNA-signaled intercommunication and reconfiguration of the two microcompartmentalized aggregated condensates is realized.

## Abstract 2

Photouncaging of compartmentalized cyanuric acid/polyadenine MDs activate transcription templates. T7 RNAp/NTPs and RNase H drive switchable/transient processes mimicking intracellular signaling and cell–cell interactions.

## Graphical abstract

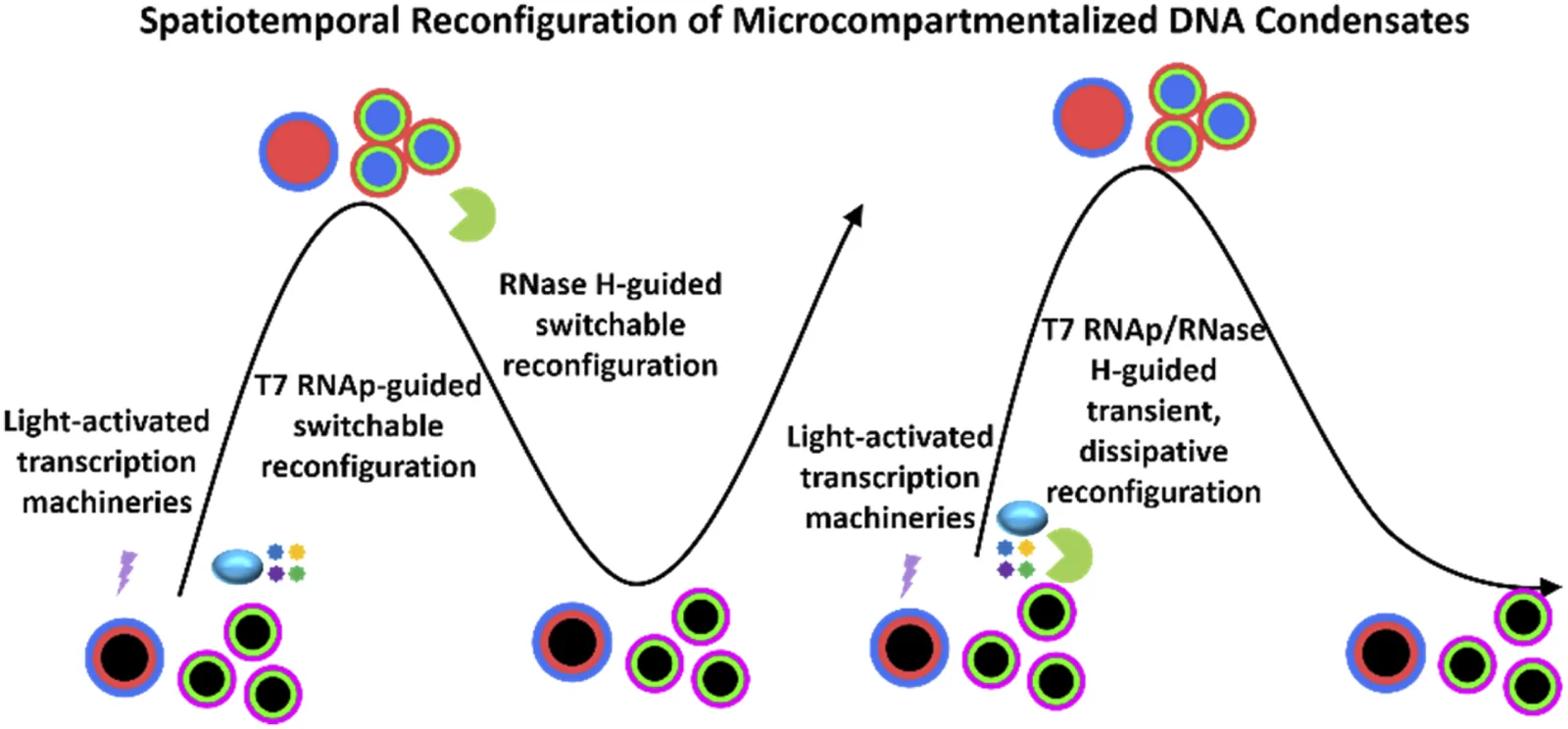

## Introduction

Mimicking native cell functions by synthetic protocell assemblies attracts substantial research interest.^1,2^ Different protocell frameworks, such as liposomes,^3–5^ polymersomes,^6–9^ proteinosomes,^10,11^ dendrosomes,^12^ colloidosomes,^13^ and hydrogel microcapsules,^14,15^ were suggested as artificial containments emulating native cells. Diverse cell-like structural and functional features were demonstrated in the protocell systems, including microcompartmentalization,^16^ self-aggregation of organelle-like structures,^17^ information transfer^18^ and signaling of the reaction pathway within the compartments.^19^ Also, the operation of programmable dynamic networks revealing signaling,^20^ cascaded,^21^ switchable,^22^ and transient catalytic transformations in the protocells^23^ were demonstrated. Moreover, fusion,^24^ differentiation,^25^ morphing^26^ and migration^27^ of protocell systems duplicating native cell functions were introduced.

Phase-separated coacervates (condensates) find growing interest as membraneless protocell reservoirs.^28^ Different materials, such as peptides,^29^ polyelectrolytes,^30^ and proteins,^31^ were employed to yield phase-separated condensates in aqueous environments. Particularly, the programmability of nucleic acid structures and their triggered reconfiguration provided versatile means to induce the emergence of phase-separated coacervates and to control their functionalities.^32–34^ Crosslinking of multi-valent Y-shaped DNA subunits^35,36^ or crosslinking of amphiphilic-DNA cross-shaped frameworks^37,38^ were implemented to induce phase-separation of DNA condensates. Integration of pH-responsive,^39^ light-responsive,^27^ enzyme responsive,^40^ or catalytic DNAzyme units^41^ into the phase-separated condensates enabled the controlled morphing structural modulation and dynamic formation and depletion of the coacervates. Furthermore, a powerful method to assemble DNA-based phase-separated condensates included coacervation of barcode functionalized cyanuric acid (CA)/polyadenine (pA) fibers into globular microdroplets (MDs).^42^ Subjecting the resulting MDs to invasive strands modified with tethers of variable complementary lengths to the barcode domains associated with the MDs resulted in the structural compartmentalization of the condensates. A competitive diffusion-hopping mechanism that dictates the compartmentalization pattern of the MDs by the complementary invasion strand lengths was introduced.^42^ While short invasive strands occupied the inner domain of the MDs, the diffusion-hopping process of long complementary invasive strands was hindered, resulting in the confinement of these strands to the exterior rim of the MDs. By using invasive strands of variable base complementarity lengths to the barcode domains, concentric compartmentalization of the MDs was demonstrated. By introducing stimuli-responsive sequences into the invading strands, the dynamic reconfiguration of the microcompartment by auxiliary triggers was demonstrated.^42^

Moreover, biocatalytic programmed transformations of DNA reaction modules have been used to synthesize phase-separated nucleic acid-based MDs and to control the properties of condensates. In this context, understanding the diffusion mechanisms associated with the spatiotemporal uptake of macromolecules by biomolecules in the phase-separated condensates and building functional MDs have attracted recent interest.^42–44^ Artificial DNA circuits in the resulting functional protocells were demonstrated.^45^ The transcription machinery, exhibiting the capacity to synthesize RNA strands encoded by the template, is an intriguing apparatus to control protocell functions and specificity in the structured DNA condensates.^46–51^ For example, barcode-modified bi-compartmentalized cholesterol-functionalized DNA-cross condensates were tethered in their inner compartment to a transcription template. In the presence of T7 RNAp/NTPs as an auxiliary catalyst, the evolved transcribed RNA exhibited the programmed sequence to be translocated to the exterior barcode-functionalized compartment, demonstrating dictated catalytic occupation of compartmentalized condensates.^43^ Also, in a recent report, a Y-shaped crosslinked DNA condensate modified with a tethered transcription template and functionalized with a fluorophore/quencher four-arm reporter was used as a coacervate to probe the transcription machinery in a confined microenvironment.^52^ In the presence of T7 RNAp/NTPs as auxiliary components, the synthesized RNA displaced the four-arm probe, and enhanced displacement within the confined reaction medium was demonstrated. The transcription machinery embedded in the condensates and the transcribed RNA product can be coupled, however, to RNase H that degrades the transcribed product. The counteracting T7 RNAp/RNase H biocatalytic process provided versatile means for spatiotemporal dynamic transformations within condensates or condensate assemblies.

Despite the progress demonstrated by the integration of transcription modules in synthetic condensates, significant challenges are still ahead of us: (i) the light-triggered spatiotemporal activation of transcription modules in pre-defined target compartments in single condensates; (ii) dynamic switchable or transient reconfiguration of the composition of compartments by transcription modules/RNase H constituents; (iii) coupling of the transcription/RNase H induced reconfiguration of the condensate composition to higher-order functionalities of the condensates such as dynamic aggregation/deaggregation process; (iv) establishing inter-condensate communication between aggregated condensate assemblies, through dictated transcription-generated RNA strands acting as information transfer strands, controlling condensate functions and behavior.

In the present study, we address these challenges by integrating dynamic photoactivated transcription modules into synthetic condensates, aiming to provide biomimetic protocell assemblies mimicking native cell functions and behavior. The condensates are microcompartmentalized by invading strands exhibiting different hybridization lengths to the barcode domains. The invading strands are pre-engineered to include photoresponsive caged structures^53^ or a transcription template. The light-triggered uncaging of the microcompartmentalized condensates leads, in the presence of auxiliary T7 RNAp/NTPs, to the activation of the transcription machinery and the transcribed RNA acts as an active signaling strand for the programmed dynamic reconfiguration of the microcompartmentalized microdroplet (MD) condensates. The light-triggered activation of the microcompartmentalized MD condensates, in the presence of T7 RNAp/NTPs and RNase H, leads to the dynamic cyclic switching and transient reconfiguration of the microcompartments. Moreover, by appropriate engineering of the microcompartmentalized, transcription machinery-embedded functional condensates, the T7 RNAp/RNase H biocatalyzed cyclic and transient aggregation/deaggregation of the condensates, accompanied by the dynamic spatiotemporal reconfiguration of the microcompartments, are demonstrated. In addition, the aggregation of two different microcompartmentalized phase-separated condensates is described, and the light-triggered reconfiguration of the condensates and transcription machinery-guided intercommunication of two different MD condensates in confined aggregate environments using a RNAp/NTP machinery are introduced. It should be noted, that previously reported^43^ transcription machinery loaded in the core of a phase-separated condensate generated a diffusive fluorescent RNA product that hybridized with a DNA constituent confined to the exterior rim domain of the droplet, thereby generating a patterned fluorescent confinement. The present study introduces, however, distinct innovative elements that advance the topic of functional transcription machinery-loaded MDs as protocell assemblies. Besides the integration of spatially confined transcription machineries into distinctly different condensates composed of cyanuric acid/polyadenine condensates using a different diffusion-hopping mechanism,^42^ the programmable functionalities introduced by spatially designed coacervate protocells represent a set of complex biomimetic transformations modelling native cells. These included signaling information transfer, switchable and transient reconfiguration of confinements and signaled cell–cell interactions and communications. The innovative, unprecedented transcription machinery guided functionalities demonstrated in the present study include: (i) the light-triggered activation of transcription machineries guiding the reconfiguration of spatially microcompartmentalized domains in the MD condensates; (ii) the introduction of programmable transcription machinery/RNase H assemblies into the microcompartmentalized condensates leading to reversible or transient dynamic reconfiguration of the microcompartment domains; (iii) designing transcription machinery/RNase H-loaded condensates demonstrating programmable switchable or transient intercommunication reflected by dynamic coacervate aggregation/deaggregation processes; (iv) designing transcription machinery-loaded condensates guiding programmed information transfer between MD condensates leading to dictated condensate aggregation and programmable microcompartment reconfiguration.

## Results and discussion

### Synthesis and microcompartmentalization of transcription machinery-loaded condensates

The preparation of the MD condensates followed the previously reported phase separation of cyanuric acid (CA) stabilized polyadenine triplex fiber coagulated condensates,^42^ functionalized with barcode (Bs1) tethers, with minor modifications, as displayed in Fig. S1. Polyadenine chains (20 nucleotides) tethered to the single strand Bs1 (pA-Bs1) were annealed in the presence of cyanuric acid in the presence of 5% polyethylene glycol (PEG-800). The resulting aqueous mixture generated barcode-functionalized condensates revealing an average size of 15 µm. (Here, PEG-800 was used as a crowding agent to expand the phase diagram, allowing the phase separation of the condensates, *cf.* Fig. S1(B) and (C). The effects of the molecular weights and concentrations of PEG, the concentrations of pA-Bs1, the concentrations of Mg^2+^ ions, and the concentrations of CA on the morphologies and structures of phase-separated MDs are displayed in Fig. S2–S6 and further discussed on p. S10). Fig. 1(A) depicts the principle of assembling transcription machinery guided compartmentalized microdroplet, MD, condensates. Treatment of the barcode-modified condensates with the transcription template T_MG_ (engineered to transcribe the malachite green (MG) RNA aptamer conjugate tethered to a barcode complementary sequence), which is conjugated to an invading blue fluorescent labeled strand, complementary to the barcode domain Bs1, yields an outer-rim blue fluorescent compartment (controlled by the length of the invading tethers). Subjecting the system to T7 RNA polymerase (T7 RNAp)/NTPs, in the presence of malachite green (MG), triggers the transcription machinery transcribing the MG RNA aptamer conjugated to the RNA tether complementary to the barcode domain. The resulting red fluorescent MG-RNA aptamer complex, tethered to the short RNA sequence complementary to Bs1, acts as a rapid invading strand that occupies the vacant inner condensate, yielding a bi-compartmentalized blue/red fluorescent condensate. The fluorescence features (red) of the synthesized MG-aptamer provide an additional fluorescent label to probe the dynamic reconfiguration of the microcompartmentalized condensate. Fig. 1(B) depicts the temporal confocal microscopy images following the dynamic formation of the bicompartmentalized MDs. It should be noted that the primary rim-confined blue condensate and the dynamically IVT-stimulated blue/red bicompartmentalized condensate follow the kinetically controlled competitive hybridization mechanism.^42^ The long complementary blue invading strand yields a kinetically arrested, rim-localized compartment (stable for five days). The IVT process does not require a Bs1 hybridization domain; however, it yields a transcribed MG RNA aptamer that includes a short complementary invading domain with Bs1, thus enabling the rapid occupation of the vacant core domain of the condensate, resulting in bicompartmentalized condensates.

**Fig. 1:**
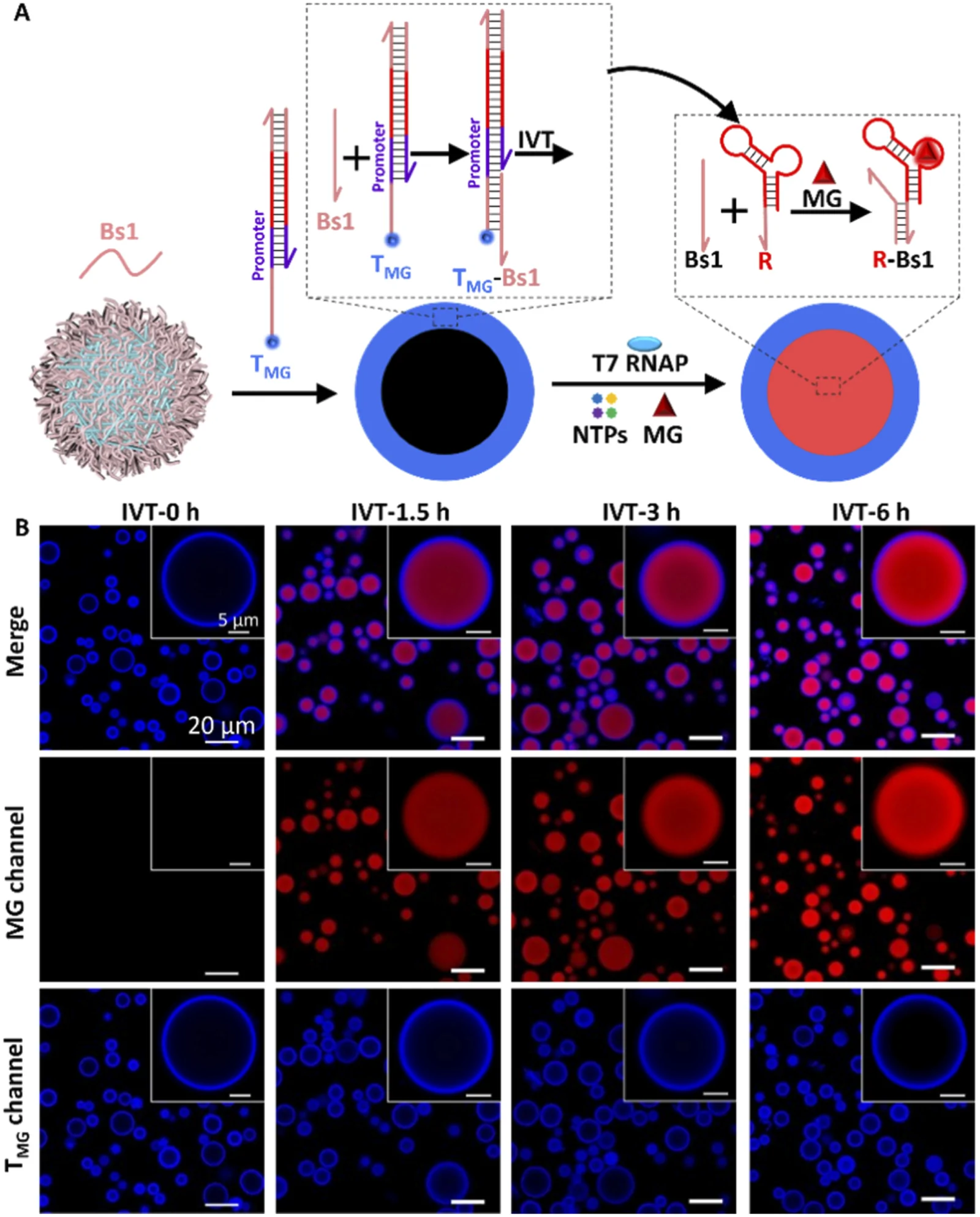
(A) Invasion of the barcode-functionalized condensates with an intact fluorophore-labeled (blue) transcription template and its activation by T7 RNAp/NTPs/MG to transcribe the MG-RNA aptamer tethered to a barcode complementary sequence leading to the dynamic invasion of the vacant core compartment by the MG-aptamer strand. (B) Time-dependent fluorescence confocal microscopy images probing the transcription machinery-guided dynamic invasion of the condensate by the transcribed RNA aptamer (scale bar = 20 µm, inset scale bar = 5 µm). Blue channel = Cy3 (*λ*_ex_/*λ*_em_ = 548/564 nm); red channel = Cy5 (*λ*_ex_/*λ*_em_ = 650/673 nm).

Subsequently, the light-triggered reconfiguration of multi-confined three-component MDs was followed by the transcription-guided dynamic reconfiguration of the MD confinements (Fig. 2). The MDs were subjected to three photoresponsive phosphate ester caged hairpins^53^ T1, T2, and S1, where T1 was modified with the blue fluorophore, T2 with the red fluorophore and S1 with an intimate green fluorophore/quencher pair. The hairpins T1, T2, and S1 included short, medium, and long binding domains to the MD barcodes. Following the competitive hybridization-diffusion invasion mechanism, the three hairpins T1, T2, and S1 generated arrested caged three-compartment MDs in state J, where T1 occupied the core domain of the MDs (blue), surrounded by the red T2 compartment and forming the non-fluorescent green/quencher S1 rim compartment. This arrested configuration of the MDs was photochemically deprotected, in the presence of the IVT/NTP transcription machinery as auxiliary constituents.

**Fig. 2:**
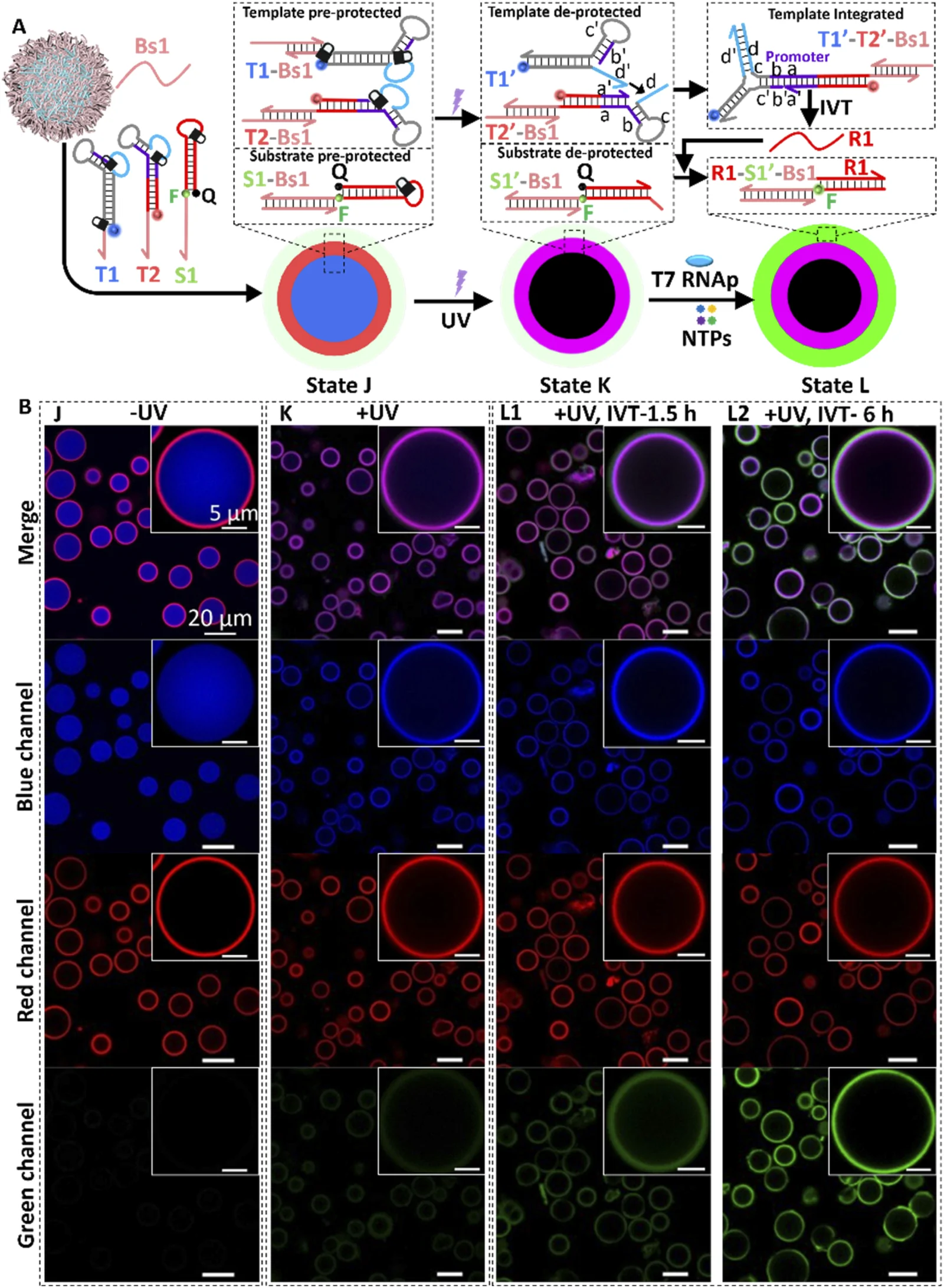
(A) Microcompartmentalization of the condensates through competitive dynamic invasion of the microdroplet with three fluorophore-labeled photoresponsive hairpins caged by *o*-nitrobenzyl phosphate ester bridging units, yielding arrested three-compartment condensates in state J. The condensates in state J are photochemically deprotected (*λ* = 365 nm) to yield a dynamically reconfigured microcompartmentalized condensate in state K, where an active transcription template emerged in a distinct compartment. Subjecting the condensates in state K to T7 RNAp/NTPs triggers the transcription machinery to transcribe an RNA strand that diffusionally displaces the quencher strand associated with the S1′-Bs1 exterior invading strand, resulting in the switched-on fluorescence of the exterior microcompartment, state L. For the detailed photoactivation of strands T1-Bs1 and T2-Bs1 to strands T1′ and T2′-Bs1 that autonomously yield the transcription template, see Fig. S10. (B) Fluorescence confocal microscopy images probing the fluorescence of the fluorophore labels associated with the microcompartmentalized condensates, using the respective fluorophore channels, in state J (prior to photo-deprotection), immediately after deprotection in state K, and at different time intervals during transcription machinery operation, allowing the RNA product-induced reconfiguration of the microcompartments in state L (scale bar = 20 µm, inset scale bar = 5 µm). Green channel = FAM (*λ*_ex_/*λ*_em_ = 493/517 nm); blue channel = Cy3 (*λ*_ex_/*λ*_em_ = 548/564 nm); red channel = Cy5 (*λ*_ex_/*λ*_em_ = 650/673 nm).

Uncaging of the blue fluorescent constituent T1 releases T1′, lacking binding to the core barcode domain. The T1′ product was pre-engineered, however, to hybridize to a photochemically uncaged constituent associated with T2′ carrying the red fluorophore and permanently linked to the barcode Bs1 associated with the core-surrounding compartment. Hybridization of T1′ with the red fluorescent compartment reorganized the middle compartment to the structure T1′–T2′-Bs1 and reconfigured the condensate to state K. This reconfiguration results in the depletion of the blue fluorescence core, and the formation of the T1′ (blue)–T2′ (red) hybrid structure yields characteristic violet fluorescence of the middle compartment, while retaining the pale green/quencher (BHQ1) feature of the exterior microcompartment (the co-localization experiment of the T1′ (blue)–T2′ (red) hybrid violet structure is displayed in Fig. S7). The reconfiguration of the hybrid constituent T1′–T2′-Bs1 compartment was pre-engineered, however, to yield a functional transcription template consisting of an intact promoter domain (b + a) linked to a transcription template. In the presence of auxiliary T7 RNAp and NTPs, the transcription machinery associated with the middle (violet) compartment is activated to transcribe the RNA product R1. The transcription template was, however, pre-designed to yield the sequence dictated transcribed product exhibiting blue complementarity to displace the quencher-modified strand, Q, associated with S1′-Bs1, leading to the reconfigured condensate in state L revealing a non-fluorescent core, surrounded by the violet T1′–T2′-Bs1 compartment and the green fluorescent compartment composed of the R1-S1′-Bs1 constituent. Fig. 2(B) depicts the confocal fluorescence microscopy images following the formation of the condensates in state J and their light-triggered reconfiguration to state K, followed by temporal transcription guided reconfiguration into state L. The high-resolution confocal fluorescence microscopy images and quantitative evaluation of fluorescence intensity changes, upon transition of the compartmentalized MDs from state J to state K, and finally to state L, are displayed in Fig. S8. Control experiments precluding the possible reconfiguration of the invaded confinement by incomplete promoter missing templates and the need to simultaneously photochemically uncage the transcription template and the caged promoter structures were performed. These experiments are presented in Fig. S9(A) and (B) with accompanying discussions. The results confirm that photo-uncaging of T1-Bs1 and T2-Bs1 is a mandatory step to stimulate the transcription involved reconfiguration of the invaded confined domains.

The photochemical deprotection of T1 yields T1′ that lacks any association with the barcodes associated with the core domain of the MDs. The pre-engineered T1′ constituents include, however, duplex and toehold domains, enabling diffusional hybridization with T2′ to yield a complete transcription template. The IVT T7 RNAp/NTP synthesis of R1 yields a functional strand displacing the quencher-labeled strand associated with S1′-Bs1, resulting in the activated green fluorescence of the S1′ compartment through separation of the quencher-modified strand from the MD framework to the bulk solution, generating compartmentalized MDs in state L.

### Phototriggered transcription machinery/RNase H mediated switchable or transient reconfiguration of microcompartmentalized condensates

We confirmed that the enzymes T7 RNAp and RNase H reveal diffusive permeation into the phase-separated condensates, Fig. S11. These features enabled the integration of T7 RNAp and RNase H as active catalytic ingredients controlling dynamic transcription machineries in the condensates. The previous example coupled the transcription machinery as a catalytic apparatus inducing the dynamic light/RNA reconfiguration of the microcompartments associated with the condensates. The integration of the added auxiliary RNase H into the system, which depletes the RNA strand, resulted in the switchable or transient, dissipative reconfiguration of the compartments. Fig. 3(A) depicts the switchable reconfiguration of the condensates. The barcode Bs1-modified condensates are challenged with the two constituents: T3, composed of the blue fluorescent complex comprising an intact promoter/template modified duplex conjugated to a long invading strand tether, and S3, composed of a photoresponsive *o*-nitrobenzyl phosphate ester hairpin structure labeled with a red fluorophore and a conjugated shorter invading strand. Invasion of the condensates with the two constituents T3 and S3, followed by washing off of the free invading strand, resulted in condensates in state M, where a void invading strand-lacking core domain is formed, surrounded by an inner condensate red fluorescent domain and an outer blue fluorescent compartment. The distinct blue/red and void (black) confinement is dictated by the length of the invading strands and the kinetic invasion mechanism.^42^ Photochemical deprotection of hairpin S3 unlocks the hairpin and yields the S3′-Bs1 duplex modified inner compartment. The subsequent treatment of the condensate with auxiliary T7 RNAp/NTPs yields the transcribed RNA R2 strand that displaces S3a′ from S3′-Bs1 while releasing S3a′. (The transcription process was conducted for 6 h). The S3a′ strand includes, however, a short invading strand, resulting in its migration into the core domain of the condensate and its reconfiguration into state N, where the core region is occupied by the red fluorescent short invading strand S3a′-Bs1 surrounded by a non-fluorescent compartment of R2-S3b′-Bs1 and an exterior blue fluorescent compartment of T3. (It should be noted that the R2 is hybridized to the S3B′-Bs1 non-fluorescent compartment and thus yields a stable hybrid compartment displayed in gray in state N). The washed-off condensates in state N were then subjected to RNase H in the buffer (0.2 U per µl), leading to the hydrolytic digestion of R2 and the S3b′-dictated dynamic extraction of S3a′ from the inner core to the S3b′-Bs1 compartment, recovering the condensate exhibiting enhanced hybridization stability of the recovered inner S3a′/S3b′-Bs1 red fluorescent compartment (hydrolysis was conducted for 3 h). Fig. 3(B) depicts the fluorescence confocal microscopy images that follow the light-induced T7 RNAp-stimulated transition of condensates in state M to state N, and the reverse RNase H-catalyzed dynamic reconfiguration of condensates in state N to state M, by probing the fluorescence features of the respective compartments through the blue/red channels of the fluorophores. The confocal fluorescence images shown in Fig. 3(B) represent a complete cycle transforming the condensate from state M to N and back. By repeated treatment of the condensates with RNAp/NTPs and RNase H and intermediate washing steps, the cycled switchable transitions between states M↔N were demonstrated (Fig. 3(C)).

**Fig. 3:**
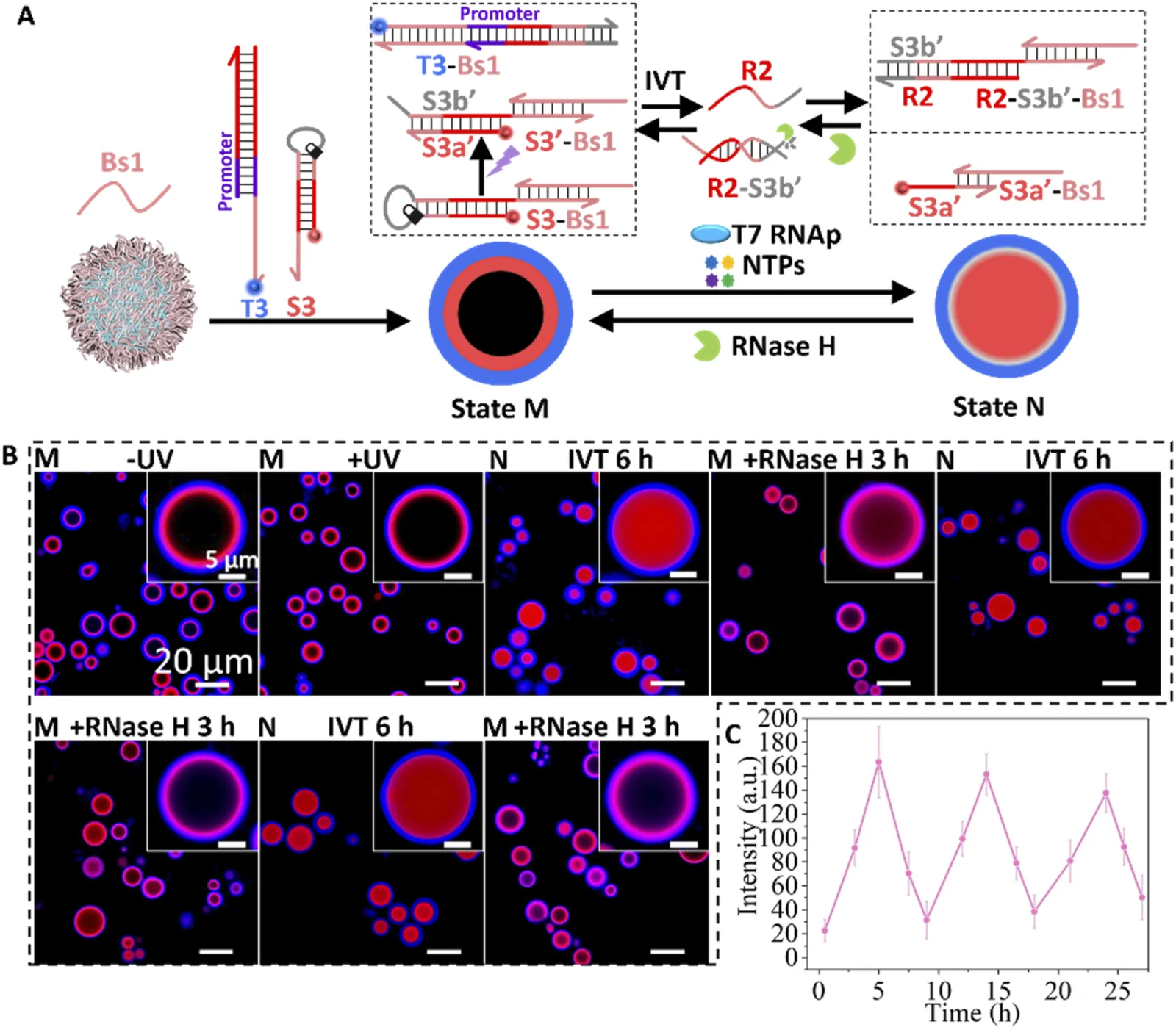
(A) Schematic switchable transcription machinery guided reconfiguration of microcompartmentalized condensates and their RNase H-catalyzed recovery. (B) Time-dependent confocal fluorescence images probing temporal switchable transitions between microcompartmentalized condensate from state M to state N and back (scale bar = 20 µm, inset scale bar = 5 µm). (C) Switchable fluorescence intensity of the red fluorescence (Cy5) core domain upon cyclic switching between State M and State N. (Error bars extracted from *N* = 3 experiments for each experiment analyzing 5 large-area 135 × 135 µm frames, with a total of 56 condensates). Blue channel = Cy3 (*λ*_ex_/*λ*_em_ = 548/564 nm); red channel = Cy5 (*λ*_ex_/*λ*_em_ = 650/673 nm).

Moreover, the system depicted in Fig. 3 demonstrated the switchable transcription-guided reconfiguration of the condensate compartments and the RNase H-induced regeneration of the condensate structure by repeated separate T7 RNAp/RNase H biocatalytic cycles. Nevertheless, one may introduce the two T7 RNAp/RNase H biocatalysts as counteracting catalysts driving the transient, dissipative, and temporal reconfiguration of the condensate compartments as outlined in Fig. 4. The condensates are invaded by the strands T3 and S3, in the presence of T7 RNAp and RNase H to yield the bicompartmentalized condensates, state M. The light-induced activation of the condensates (*λ* = 365 nm) and the concomitant addition of the NTP bases activate the transcription machinery and the temporal reconfiguration of the condensates to the intermediate structure consisting of state N. The presence of RNase H degrades, however, the duplex R2/S3b′, resulting in the counter depletion of the intermediate condensate structure, recovering the original condensate compartmentalized configuration. Nevertheless, the design of such a transient temporal reconfiguration of the condensate structure is anticipated to demand a delicate concentration balance of the counteracting biocatalysts, T7 RNAp/RNase H. Fig. 4(B) depicts the temporal confocal fluorescence images of the condensates (probed by following the blue/red channels), upon operating the transient formation of the intermediate state N condensates and their recovery to state M. Evidently, after *ca.* 6 h, the intermediate condensates in state N reach a peak value and then level off with full recovery of state M after 15 h. Control experiments (Fig. S12–S17) confirmed the modulated balance between the concentrations of T7 RNAp and RNase H. While low concentrations of RNase H prevented the transient reconfiguration process, high RNase H concentrations prevented the formation of the condensates in transient state N. Fig. 4(C), panel I, depicts the temporal transient fluorescence changes (red channel) upon the formation of reconfigured state N and its recovery to state M, using different T7 RNAp/RNase H ratios, whereas panel II depicts control experiments. As the concentration of RNase H decreases (fixed concentrations of T7 RNAp and NTPs), the recovery of state M is prolonged. For the related temporal fluorescence changes, in the presence of different RNase H concentrations, and the temporal fluorescence changes observed in the control systems, see Fig. S12–S17, SI. Moreover, the transient transcription efficiency and accompanying microcompartment reconfiguration as a function of NTP concentrations, at fixed T7 RNAp/RNase H concentrations, were examined. The results are presented in Fig. S18. As the concentration of NTPs increased, the peak concentrations of the transcribed RNA increased, and the depletion time intervals of the transient processes were prolonged. Phototriggered transcription machinery/RNase mediated switchable or transient concomitant reconfigured microcompartmentalization and aggregation/deaggregation of condensates.

**Fig. 4:**
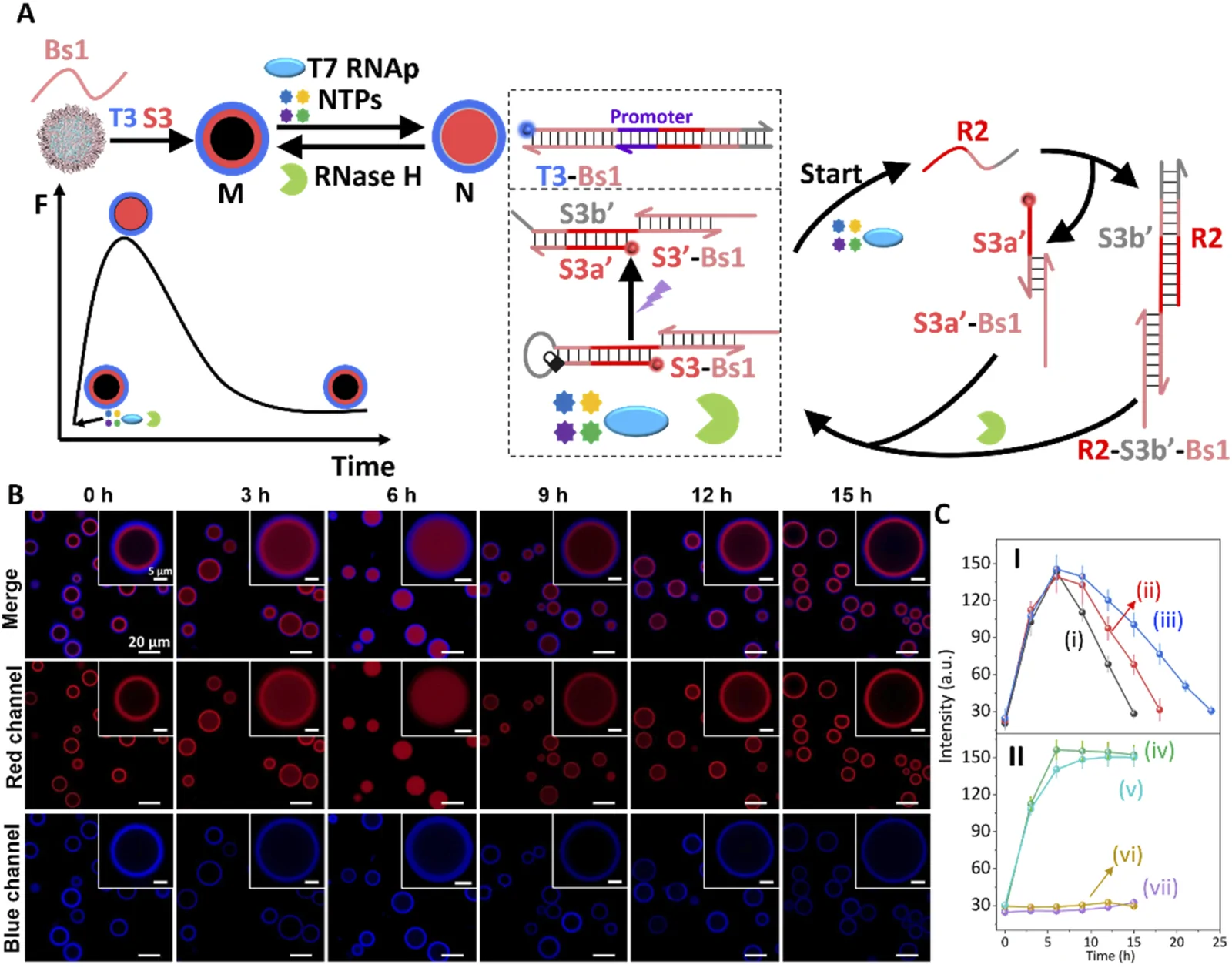
(A) Transient transcription machinery guided transition between state M to state N in the presence of T7 RNAp/NTPs and RNase H. (B) Time-dependent fluorescence images probing the transient transitions of condensates in state M to state N following the fluorescence feature of the fluorophore associated with the microcompartmentalized condensates (scale bar = 20 µm, inset scale bar = 5 µm). (C) Panel I, transient fluorescence intensities of the red fluorophore (Cy5) upon dynamic transient transition between state M and state N and back in the presence of different T7 RNAp: RNase H ratios: (i) 200 : 1; (ii) 300 : 1; (iii) 500 : 1. (T7 RNAp 2 U per µl and RNase H in (i) 0.01 U per µl (ii) 0.0067 U per µl (iii) 0.004 U per µl). Panel II, control experiment following the temporal fluorescence intensities of the red fluorescence of Cy5 in the T7 RNAp/RNase H machinery system: (iv) in the absence of RNase H; (v) in the presence of low RNase H concentration T7 RNAp: RNase H 1000 : 1 (T7 RNAp 2 U per µl; RNase H 0.002 U per µl); (vi) in the absence of T7 RNAp; (vii) in the presence of high RNase H concentration, T7 RNAp: RNase H 100 : 1 (T7 RNAp 2 U per µl; RNase H 0.02 U per µl). (Error bars extracted from *N* = 3 experiments for each experiment analyzing 5 large-area 135 × 135 µm frames, with a total of 62 condensates) green channel = FAM (*λ*_ex_/*λ*_em_ = 493/517 nm); blue channel = Cy3 (*λ*_ex_/*λ*_em_ = 548/564 nm); red channel = Cy5 (*λ*_ex_/*λ*_em_ = 650/673 nm).

The concept introduced in Fig. 3 and 4 demonstrating the switchable or transient reconfiguration of the compartments associated with the condensates by means of an auxiliary T7 RNAp/NTPs and RNase H machinery was further extended by designing functional condensates enabling triggered switchable or transient aggregation/deaggregation of the condensates accompanied by the concomitant reconfiguration of the condensate compartments. Fig. 5(A) depicts schematically the assembly of microcompartmentalized condensates in the presence of T7 RNAp/NTPs and RNase H as auxiliary machinery, the light triggered switchable aggregation/deaggregation of the condensates, and the dynamic reconfiguration of the condensate microcompartments. The separated condensates in state O were synthesized by invading strands T4 consisting of the medium length invading domain tethered to a green labeled promoter/transcription template, and the caged *o*-nitrobenzyl phosphate ester hairpin strand tethered to the long invading domain and modified with the Cy3/Cy5 FRET fluorophore pair. Following the kinetic invading principle,^42^ the condensates exhibited the compartmentalization shown in state O, where a vacant core compartment is surrounded by a green T4-Bs1 compartment and an exterior rim compartment consisting of a composite fluorescent microcompartment (displayed as “magenta”) that represents the overlay of red Cy5 (Cy5 *λ*_ex_ = 640 nm, Cy5 *λ*_em_ = 668 nm) and FRET generated by the constituent S4-Bs1, where FRET between Cy3 and Cy5 proceeds (Cy3 *λ*_ex_ = 561 nm, maximum FRET signal *λ* = 659 nm appearing as violet). Photochemical uncaging of the condensate in state O unlocks the S4-Bs1 hairpin compartment to S4′-Bs1, and in the presence of added T7 RNAp/NTPs, the transcription machinery is activated to yield the RNA product R3. The transcription template and the uncaged S4′ constituent are pre-engineered to allow the displacement of the D strand associated with the S4′-Bs1 constituent by R3, yielding the R3-D duplex. The transcribed R3, besides exhibiting complementarity to the D strand sequence, is conjugated to a short nucleic acid tether acting as an invading strand complementary to the barcode units Bs1. As a result, the ongoing transcriptions and the formation of R3 introduce complementary functions into the duplex R3-D reconfiguring the condensates in state O to condensates in state P. In state P, the blue-labeled duplex R3-D modified with the short invading domain is dynamically translocated to the interior core compartment, which is surrounded by the green T4-Bs1 compartment, and retains the red fluorophore A(Cy5)-Bs1 constituent comprising the exterior rim compartment of the condensates. The transcription machinery stimulated separation of S4′-Bs1 yields the free A(Cy5) tethered constituent as a functional constituent of the exterior compartment. Nevertheless, A was pre-engineered to include a palindromic domain f. These self-complementary sequences associated with the exterior compartment enabled the inter-hybridization of condensates and their autonomous temporal aggregation. The subsequent treatment of the aggregated condensates in state P with RNase H leads, however, to the digestive hydrolysis of R3 and the release of strand D in the core domain. The released blue-labeled D lacking any binding interactions with the core barcode units diffuses across the condensates leading to the separation of the A–A self-complementary bridging units by forming the original S4′-Bs1 duplex exhibiting enhanced base-pairing stability. The RNase H-stimulated reaction leads to the separation of the aggregated condensates and the recovery of the condensates in state O. By repeated treatment of the condensates in state O with T7 RNAp and RNase H, while washing off the biocatalysts within their alternate operation, the cycled switchable reconfiguration and aggregation/deaggregation of the condensates is anticipated to proceed. Fig. 5(B) depicts the cyclic stepwise light-triggered, transcription-guided reconfiguration of condensates in state O to aggregated condensates of state P and RNase H-stimulated deaggregation of condensates in state P. Temporal structures are probed by the merged fluorescence image (upper entry) and FRET fluorescence channel (bottom entry) along the cyclic operation of the respective constitutional structures. (For the experimental procedure employed to induce the switchable cyclic transcription machinery guided aggregation of the condensates in state O to state P, and the subsequent RNase H-stimulated separation of the aggregates, see the SI, Experimental Section 1.5). Fig. 5(C) shows the switchable OFF-ON FRET fluorescence intensities upon operating three successive T7 RNAp/RNase H reconfigured aggregation/deaggregation cycles of the condensates. Fig. 5(D) depicts the statistical analysis of the assembly counts upon driving three repeated switchable IVT/RNase H aggregation/deaggregation cycles. (Assembly count = number of MDs in an aggregate). Evidently, the average assembly corresponding to *ca.* 16 MDs in an IVT generated aggregate is observed, whereas upon RNase H treatment the aggregates are fully separated. (The results present statistical analysis of *N* = 3 different experiments, each experiment with 8 large-area 135 × 135 µm frames).

**Fig. 5:**
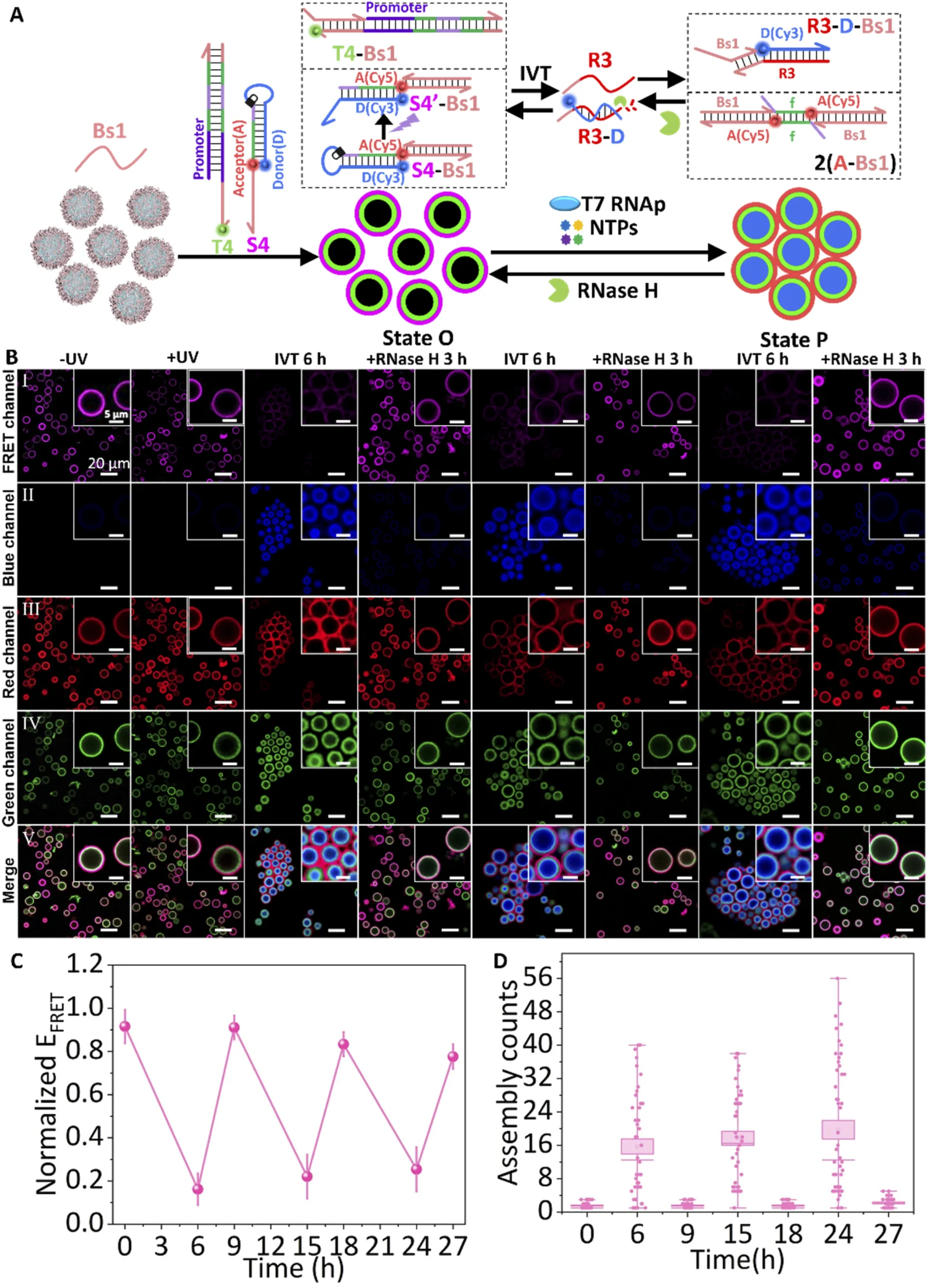
(A) Schematic transcription machinery/RNase H guided switchable aggregation/deaggregation of microcompartmentalized condensates. The parent condensates consist of competitively invaded microcompartmentalized condensates in state O composed of an inner void (empty) core surrounded by the green fluorescent compartment occupied by the T4 transcription template and the exterior violet fluorescent microcompartment composed of the FRET invading constituent S4. Photodeprotection of S4 followed by the triggered T7 RNAp/NTP transcription of strand R3 results in the reconfiguration of the condensates in state O into the aggregated condensates in state P. For the detailed stepwise reconfiguration of condensates in state O to aggregates in state P, see text. The reconfiguration of the microcompartments involves the occupation of the core with the R3-D invader surrounded by the T4 constituent microcompartment and a rim red fluorescent microcompartment of constituent A. The self-complementary palindromic domain of A drives the aggregation of the condensates. Subsequent addition of RNase H leads to the reverse deaggregation of state P to state O, while recovering the original composition of the microcompartments in state O. (B) Temporal confocal microscope images corresponding to the fluorescence features of the microcompartmentalized domains upon the triggered dynamic switchable reconfiguration of the microcompartments of state O to state P and the reverse RNase H-stimulated recovery of state O probed in the rows of panels I to V through the FRET channels (*λ*_ex_/*λ*_em_ = 548/673 nm), red channel (*λ*_ex_/*λ*_em_ = 650/673 nm) and green channel (*λ*_ex_/*λ*_em_ = 493/517 nm). Row I: temporal depletion and regeneration of the FRET fluorescence (*λ*_ex_/*λ*_em_ = 548/673 nm) associated with S4-Bs1 or S4′-Bs1 in the exterior microcompartment. Row II: temporal evolution of the blue fluorescent aggregates of condensates (*λ*_ex_/*λ*_em_ = 548/564 nm) and their subsequent deaggregation accompanied by the depletion of the blue fluorescence; Row III: temporal red fluorescence of Cy5 (*λ*_ex_/*λ*_em_ = 650/673 nm) in the exterior microcompartment showing transition from the separated condensates to aggregated condensates (after 6 h) followed by deaggregation of the condensates upon addition of RNase H along a time interval of 3 h. Row IV: green fluorescence of FAM (*λ*_ex_/*λ*_em_ = 493/517 nm) in middle compartment of the condensates. Row V: temporal merge images (scale bar = 20 µm, inset scale bar = 5 µm). (C) Cyclic integrated FRET intensities of the exterior microcompartment, probing three temporally consecutive cycles of IVT activation of the transcription machinery at *t* = 6 h, 15 h, and 24 h, followed by three RNase R3 digestion cycles by RNase H after *t* = 9 h, 18 h and 27 h; (D) statistical analysis of the assembly counts (corresponding to the number of MDs in the different aggregates upon the switchable IVT/RNase H aggregation/deaggregation cycles). Temporal IVT (6 h, 15 h, and 24 h). Temporal RNase depletion cycle (9 h, 18 h, and 27 h). (Error bars extracted from *N* = 3 experiments for each experiment analyzing 10 large-area 135 × 135 µm frames, with a total of 52 condensates).

We then extended the concept of T7 RNAp/NTPs and RNase H guided switchable aggregation/deaggregation to develop a transient aggregation/deaggregation operating system. Fig. 6 depicts schematically the transient transcription machinery guided aggregation/deaggregation of the condensates accompanied by the transient fluorescence changes occurring in the respective microcompartments. In this system, the parent microcompartmentalized condensates are subjected, in parallel, to the two enzymes T7 RNAP/NTPs and RNase H. The parent condensates include the green fluorescent compartment of T4-Bs1 and the magenta of fluorescent compartment of S4-Bs1. Photochemical uncaging of the S4-Bs1 is followed by the transcription machinery guided displacement of the blue fluorescent strand D, its hybridization with the transcribed R3 and translocation of blue D(Cy3)/R3 duplex to occupy the core compartment of the condensates. The blue core surrounded by the green compartment, composed of the T4-Bs1 constituent and the red-labelled A(Cy5) constituent comprises the exterior rim compartment composing the three compartment condensates. The palindromic domain f associated with strand A, composing the exterior compartment, results in the aggregation of the condensates into state P. The coexistence of RNase H in the system leads, however, to the cleavage of R3 and the release of the blue-labelled constituent, lacking any affinity towards the core region of the condensates. The diffusional migration of the blue-labelled strand D, across the condensates leads to the energetically stabilized S4′-Bs1 structure comprising the exterior rim constituent, resulting in the separation of the f/f bridged linking aggregated condensates and the recovery of the parent condensates in state O. That is, subjecting the condensates in the configuration of state O to the mixture of the transcription machinery T7 RNAp/NTPs and RNase H leads to the competitive transcription-guided aggregation of the condensate and the RNase H-induced transient dissociation of the aggregates, in state P, to the parent condensates in state O. Fig. 6(B) depicts the temporal fluorescence changes and the degree of condensate aggregation probed through the FRET (Cy5, *λ*_ex_/*λ*_em_ = 548/673 nm), the blue fluorescent channel (Cy3, *λ*_ex_/*λ*_em_ = 548/564 nm) and the red fluorescent channel (Cy5, *λ*_ex_/*λ*_em_ = 650/673 nm). The respective fluorescent intensities and aggregation decrease/increase and reach peak values after a time interval of 6 h and afterwards temporally decrease in intensity, reaching the original fluorescence pattern associated with the parent rest state of the condensates. These temporal fluorescence aggregation/deaggregation features are consistent with the transient and dynamic reconfiguration feature outlined in Fig. 6(A). The green fluorescent channel reveals, however, a constant fluorescence intensity throughout the entire time interval of operating the process within the condensates. This result is consistent with the fact that the green fluorescent labeled transcription template T4-Bs1 stays positionally intact within the condensate framework (middle compartment that is not affected by all other dynamic constituents). The temporal transient fluorescence intensity changes accompanying the temporal transcription/RNase H-induced reconfiguration of the compartment and of the dynamic aggregation/deaggregation of the condensates are expected to be controlled by the ratio of T7 RNAp/RNase H controlling the dynamic process. The temporal fluorescence changes through the respective probing channels, in the condensate system shown in Fig. 6(B), were recorded at a T7 RNAp/RNase H ratio corresponding to 200 : 1. Similar experiments were performed at biocatalyst T7 RNAp/RNase H ratios corresponding to 300 : 1 and 500 : 1 and the results are displayed in Fig. S19 and S20. Fig. 6(C) depicts the transient temporal FRET channel responses for the three T7 RNAp/RNase H systems (curves i, ii, iii). The FRET channel response demonstrates an initial depletion of the FRET signal followed by the temporal recovery to the original value. While the time interval for reaching the minimum FRET intensity is similar for all systems, *ca.* 6 hours, the recovery of the parent FRET signal is prolonged as the relative concentration of RNase H decreases. The primary decrease in the FRET signal is consistent with the separation of the complex S4′-Bs1 by R3 and the recovery of the FRET signal is due to the RNase H catalyzed cleavage of R3 and the recovery of the S4′-Bs1 constituent. Similarly, curves (i), (ii), and (iii) present the transient fluorescence changes observed in the blue fluorescent channel upon operating the three systems. The temporal blue fluorescence intensities initially increase to a peak value after *ca.* 6 hours, yet the regeneration of the parent fluorescent state is affected by the concentration of RNase H and is prolonged as the RNase concentration decreases. Fig. 6(D) depicts the temporal aggregation counts (number of condensates in aggregated structures per frame, averaged over frames at each time interval), for the transiently operated T7 RNAp/RNase H-driven aggregation/deaggregation system at biocatalyst ratios corresponding to (a) 200 : 1, (b) 300 : 1 and (c) 500 : 1. In all three systems, peak average aggregation counts for the aggregated structure are observed after *ca.* 6 h, yet the decay times for deaggregation of the structure are influenced by the concentration of RNase H, and as the concentration of RNase H is lower, the time of regeneration of the parent fully deaggregated condensates is longer. For example, at a T7 RNAp : RNase H ratio of 200 : 1, the recovery of the parent individual condensates is *ca.* 9 h, whereas the recovery time of the separated condensates at a biocatalyst ratio of 500 : 1 is 18 h. For the related temporal transient fluorescence changes, in the presence of different T7 RNAp : RNase H concentrations, see Fig. S19 and S20, SI.

**Fig. 6:**
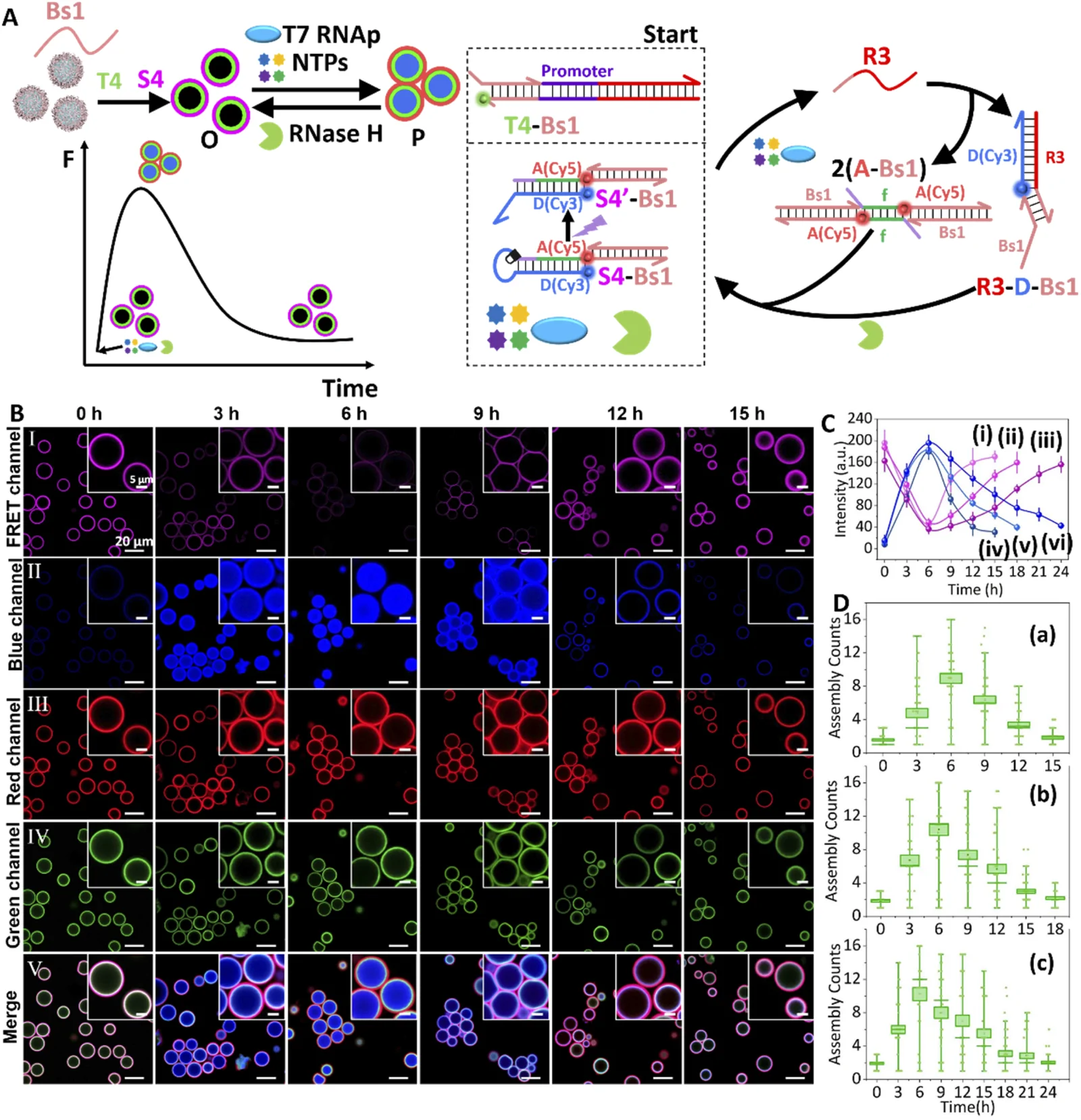
(A) Schematic light-triggered, transient, transcription machinery-guided reconfiguration of the microcompartmentalized constituents of state O to state P and back, accompanied by aggregation/deaggregation of the condensates, in the presence of T7 RNAp/NTPs and RNase H (for the composition of the constituents and detailed operation of the transient reconfiguration processes, see Fig. 5(A)). (B) Temporal fluorescence changes associated with the condensates of the transiently reconfigured microcompartmentalized domain associated with the dynamic transition between state O and state P and back, accompanied by the transient aggregation/deaggregation of the condensates, probed by the FRET signals of the exterior microcompartment of S4′-Bs1, the blue transient fluorescence of the R3-D-Bs1 strand occupying the core domain of the condensate and the green fluorescent domain occupied by the transcription template T4, separating the core domain and the exterior microcompartment (scale bar = 20 µm, inset scale bar = 5 µm). Green channel = FAM (*λ*_ex_/*λ*_em_ = 493/517 nm); blue channel = Cy3 (*λ*_ex_/*λ*_em_ = 548/564 nm); red channel = Cy5 (*λ*_ex_/*λ*_em_ = 650/673 nm), and FRET channel = (*λ*_ex_/*λ*_em_ = 548/673 nm). (C) Transient FRET responses of the exterior compartment upon the dynamic transitions between state O to state P and back, in the presence of different ratios of T7 RNAp/RNase H: (i) 200 : 1, (ii) 300 : 1, and (iii) 500 : 1, and transient integrated blue fluorescence intensities of the R3-D-Bs1 constituent, in the presence of variable ratios of T7 RNAp/RNase H: (iv) 200 : 1, (v) 300 : 1, and (vi) 500 : 1. (D) Temporal average aggregation counts of the condensates (number of condensates in aggregated structures per frame monitored at each time interval analyzing 10 frames), in the presence of variable T7 RNAp/RNase H ratios: (a) 200 : 1, (b) 300 : 1, and (c) 500 : 1. (Error bars extracted from *N* = 3 experiments for each experiment analyzing 10 large-area 135 × 135 µm frames, with a total of 58 condensates).

### Programmed transcription machinery-regulated reconfiguration of microcompartments within intercommunication aggregated condensates

Moreover, an auxiliary transcription machinery-guided intercommunication within microdroplet aggregates consisting of two microcompartmentalized MDs W and V, leading to the reconfiguration of the compartments in the aggregates, was realized (Fig. 7). Two cyanuric acid/pA condensates functionalized with complementary barcode tethers, Bs1 and Bs1′, W and V, are used to generate the W/V aggregates. Reacting the aggregates with three *o*-nitrobenzyl phosphate ester caged hairpin structures T1, T2 and S5, where T1 and T2 include recognition tethers for Bs1 and strand S5 includes a recognition tether for Bs1′, are used for the competitive invasion and microcompartmentalization of the respective condensates in the aggregated framework. T1 and T2, exhibiting short and long invading tethers complementary to Bs1, and strand T1 are labeled with a blue Cy3 fluorophore and T2 is labeled with the red Cy5 fluorophore. Thus, the guided compartmentalization of condensates W with a blue fluorescent core, surrounded by a red fluorescent rim, evolves upon competitive invasion of condensates W. Strand S5 modified with a long invading tether complementary to Bs1′ and functionalized with a fluorophore (FAM)/quencher (BHQ1) pair invades the condensates V to yield a weak green fluorescent rim of compartmentalized condensates V. Phototriggered uncaging of the protected hairpins in the two condensates leads to a free T1′ constituent in the core domain of W and to an uncaged T2′-Bs1, the red fluorescent constituent in the rim domain. Photouncaging of S5 in condensate V leads to a weak fluorescent S5′-Bs1′ duplex constituent comprising the rim domain of V. The diffusional blue fluorescent constituent T1′ is pre-designed to migrate into the rim domain, where hybridization with T2′-Bs1 proceeds, yielding a violet fluorescent (blue + red) rim and leaving a non-fluorescent core. The constituent T1′-T2′-Bs1 in condensate W is pre-designed to act as a transcription template. Thus, in the presence of T7 RNAp/NTPs, as an auxiliary catalytic module, transcription proceeding in condensate W yields the RNA product R4. The diffusional product R4, lacking any binding affinity to MDs W, is, however, pre-engineered to be diffusionally translocated to condensates V in the aggregates, and its hybridization capacities displace the duplex S5′ to yield R4-S5′-Bs1 as functional constituents. Displacement of the quencher-modified strand resulted in a reconfigured rim associated with condensate V, exhibiting green fluorescence. Thus, the transcribed strand R4 acts as an inter-communicating strand reconfiguring the microcompartments associated with V. Fig. 7(B) displays the confocal fluorescence microscopy images associated with the dynamic reconfiguration of the compartments associated with condensates W and the transcription-guided reconfiguration of condensates V. The invaded aggregates consisting of condensates W and V in state X reveal the blue core fluorescence and the red rim fluorescence of condensate W and weak green fluorescence associated with the fluorophore-quenched rim associated with condensate V. Phototriggered uncaging of the invading hairpins leads to state Y, where the core constituents in condensates W diffuse to the rim domains, leading to violet fluorescence comprising merged blue and red fluorescence. As the transcription process proceeds, the green fluorescence associated with condensates V in the aggregates evolves, confirming the translocation of strand R4 from condensates W to V and displacement of S5′, resulting in state Z. (For further support confirming the Bs1/Bs1′ complementary bridging of the aggregated MDs W/V, see Fig. S21 and accompanying discussions. The 3D-stack confocal fluorescence microscopy image of light/transcription machinery-generated aggregated MD W/V in state Z is displayed in Fig. S22. For the enhanced transfer and RNA strand intercommunicated reconfiguration in the aggregated condensates W/V, as compared to the separated condensates W′/V′, see Fig. S23 and accompanying discussion.) It should be noted that despite the complementarity of the barcode strands associated with MDs W and V that assisted the aggregation of the condensates, no inter-condensate permeation and fusion could be observed for a time interval of 10 days (Fig. S24). Presumably, the negative charge associated with the MDs provides an electrostatic kinetic barrier preventing the fusion process.

**Fig. 7:**
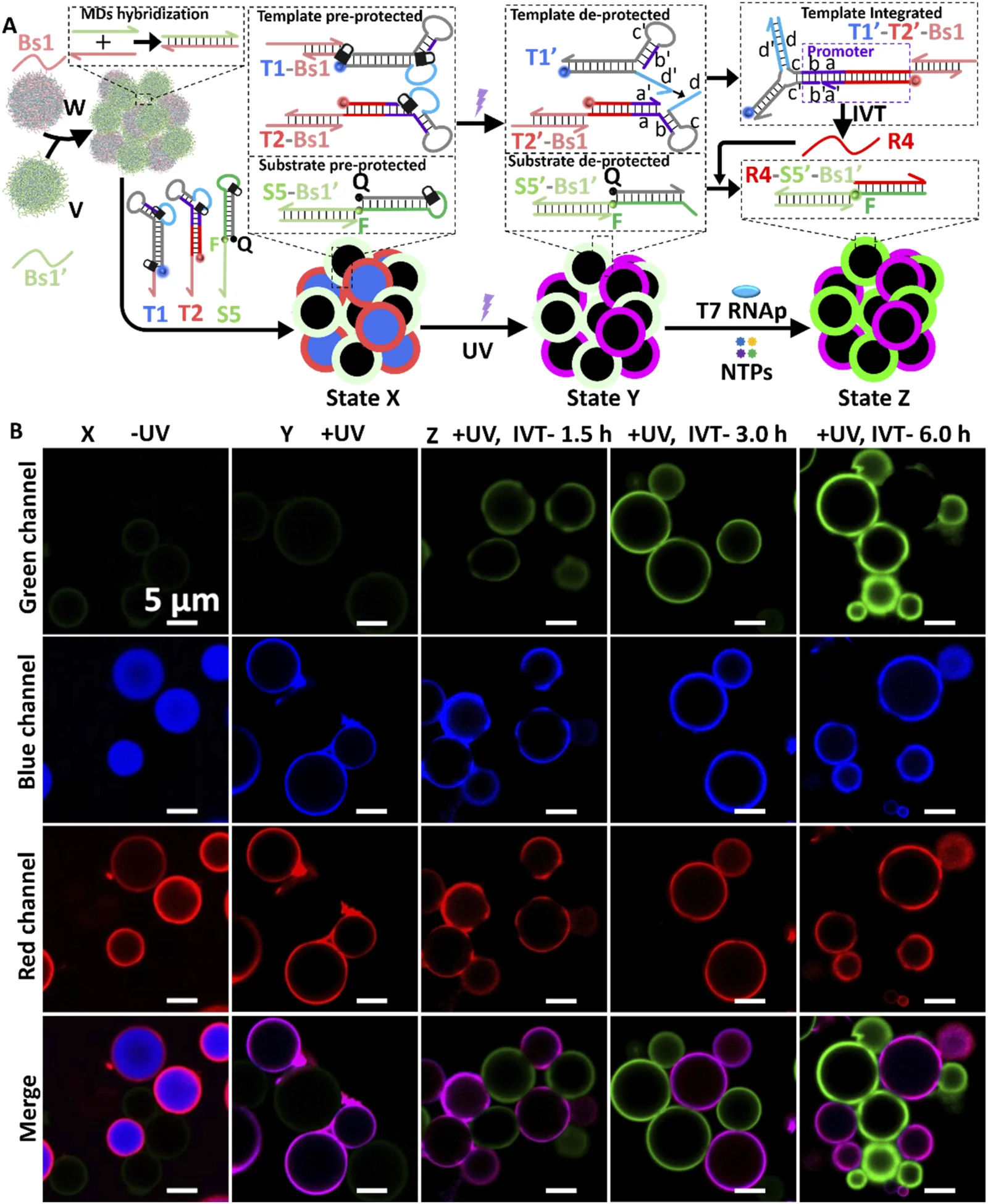
(A) Schematic aggregation of two microcompartmentalized condensates W/V, yielding state X. Photochemical activation of the microcompartmentalized aggregated condensates in state X leads to reconfigured aggregated condensates in state Y, undergoing a temporal transition to state Z, in the presence of an auxiliary T7 RNAp/NTPs machinery. (B) Confocal microscopy images probing the fluorescence features of the aggregated microcompartmentalized condensates: before photochemical uncaging, state X, after photochemical uncaging, state Y, and upon temporal T7 RNAp/NTP-stimulated transition of state Y to state Z. Top to bottom entry: green channel, blue channel, red channel and merged images of the condensates in the respective states and temporal transition of state Y to state Z (scale bar = 20 µm, inset scale bar = 5 µm). Green channel = FAM (*λ*_ex_/*λ*_em_ = 493/517 nm); blue channel = Cy3 (*λ*_ex_/*λ*_em_ = 548/564 nm); red channel = Cy5 (*λ*_ex_/*λ*_em_ = 650/673 nm).

The barcode-mediated aggregation of condensates W and V and the light-triggered transcription-guided reconfiguration of the condensates by the transcribed R4 strand revealed enhanced intercommunication efficiency in the aggregated assembly as compared to the separated condensate configuration. This is due to the intimate, high-concentration channelling of R4 in the aggregated assembly, as compared to a diffusive, random, low concentration of R4, interlinking the separated condensates. The system depicted in Fig. 7 is, however, irreversible (neither switchable nor dissipative). Designing future stimuli responsive complementary barcode bridges *e.g.* photoresponsive azobenzene-modified duplex^54^ barcodes or ligand/aptamer complexes that are catalytically depleted (*e.g.* adenosine/adenosine deaminase)^55^ could transform these structurally modulated aggregates into reversible or transient systems.

## Conclusions

Membraneless organelles consisting of phase-separated nucleic acids or protein condensates are key constituents controlling cell functions.^56–58^ The organelles composed of compact compartmentalized microenvironments regulate diverse physicochemical cell functions.^59,60^ For example, organelles such as nucleoli, Cajal bodies and stress granules regulate biogenetic transcription and posttranscription processes^61–63^ and inter-organelle signaling plays central roles in controlling cellular transformations.^64,65^ Our study introduced biomimetic approaches to model native organelle functions by synthetic phase-separated condensates. Specifically, we demonstrated the integration of caged transcription templates in the microcompartmentalized microdroplet condensates, their light-induced uncaging, and the triggered T7 RNAp/RNase operation of transcription machineries, resulting in the switchable or transient reconfiguration of the condensate microcompartments by temporally generated RNA strands. Moreover, by pre-engineering the condensate structure or pre-engineering the structure of a mixture of condensates, the transcription guided switchable or transient aggregation/deaggregation of the condensates was demonstrated. Besides, the intra-condensate reconfiguration of the compartmentalized microenvironments upon aggregation/deaggregation of the droplets and the transcription-regulated intercommunication and signaling programmed reconfiguration of microcompartments within the aggregates were introduced. Beyond the mimicking features of native organelles, the study advanced the area of systems chemistry by establishing synthetic transcription machinery-assisted regulated switchable, transient and signaling structural reconfiguration of cell-like containments.

While important advances emulating cell functions by synthetic phase-separated condensates were presented, significant challenges are ahead of us. Besides structural reconfiguration of the condensate microstructures by auxiliary catalysts (enzymes), the integration of catalysts within the phase-separated condensates and executing programmed autonomous functions by auxiliary agents, such as ions, light or metabolites, is desirable. For example, integration of DNAzymes into phase-separated condensates,^39^ their triggered activation by light, ions or amino acid cofactors toward programmed catalytic reactions or microdroplet replication can be envisaged.

## Author contributions

IW, FX, and FH conceptualized and supervised the project. SX and YQ formulated and designed the experiment. SX, YQ, YH and YW participated in the experiments. IW, FX, FH, SX, YQ, YH and ZT participated in writing the paper and its editing.

## Conflicts of interest

The authors declare no competing financial interests.

## Supplementary Material

SC-OLF-D6SC06136B-s001

## Data Availability

The data supporting this article have been included as part of the supplementary information (SI). Supplementary information: Experimental section, detailed DNA sequences, supporting figures and accompanying discussions. See DOI: https://doi.org/10.1039/d6sc06136b.
