# Supplementary material for: Light-activated, transcription-guided reconfiguration, aggregation, and intercommunication of structurally compartmentalized DNA condensates
Source: Chem Sci. 2026 Oct 30. Online ahead of print. doi: 10.1039/d6sc06136b (PMC13721794; doi:10.1039/d6sc06136b)
Supplement: Supplementary file 1 [file SC-OLF-D6SC06136B-s001.pdf]

Supplementary Information (SI) for Chemical Science.
This journal is © The Royal Society of Chemistry

Supporting Information for

Light-Activated, Transcription-Guided, Reconfiguration, Aggregation, and Intercommunication of Structurally Compartmentalized DNA Condensates

Shijun Xu[1,#], Yichen Han[2,#], Yunlong Qin[2,#], Zhenliang Tian[1], Yunxiao Wang[1], Itamar Willner[2], Fan Xia*[1], and Fujian Huang*[1]*

[1] State Key Laboratory of Geomicrobiology and Environmental Changes, Faculty of Materials Science and Chemistry, China University of Geosciences, Wuhan 430074, China. Email: xiafan@cug.edu.cn; huangfj@cug.edu.cn

[2] The Institute of Chemistry, The Hebrew University of Jerusalem, Jerusalem 91904, Israel. Email: itamar.willner@mail.huji.ac.il

[#] These authors contributed equally.

1. Experimental section

1.1 Materials

Cyanuric acid (CA) was procured from Sa'en Chemical Technology Co., Ltd. Malachite Green (MG) was sourced from Maclean. Sodium chloride (NaCl), magnesium chloride ($MgCl_2$), and polyethylene glycols with diverse molecular weights utilized in the present experiment were obtained from Sinopharm Chemical Reagents Co., Ltd., China. T7 RNA Polymerase, nucleoside triphosphates (NTPs), and Ribonuclease H (RNase H) were

purchased from New England Biolabs (NEB). All chemical reagents employed in this experimental study were of analytical grade and did not require further purification. All nucleic acid sequences were obtained from Hippo Biotechnology Co., Ltd. (Beijing, China) and were purified using high-performance liquid chromatography (HPLC). The detailed nucleic acid sequences are illustrated in Tables S1 - S3. The concentrations of all nucleic acid sequences were evaluated by the absorbance at the wavelength of 260 nm using a UV-vis spectrometer.

1.2 Preparation and Purification of DNA microdroplets

An optimized protocol for the preparation of cyanuric acid/polyadenine (CA/pA) DNA microdroplets (MDs) was established based on our previous study. In a typical protocol, unless otherwise mentioned, a mixture (30 μL) composed of 5% (w/w) PEG-800, 6 mM CA, 20 mM $MgCl_2$, and 10 μM pA – Bs was prepared in a 200 μL Eppendorf tube. The samples were heated in a dry bath (Labnet International D1100 Accublock Digital) at a temperature of 95°C and incubated for 30 min. Subsequently, the samples were gradually cooled from 95°C to room temperature over a period of 2 h, resulting in phase-separated cyanuric acid/polyadenine crosslinked MDs. In addition, the effects of PEG as a crowding agent (degree of polymerization, concentration), the effects of pA concentration, the effects of Mg^{2+} concentration, and the effects of CA concentration on the morphology of phase-separated MDs were investigated and optimized. To eliminate possible unreacted components, the resulting MDs were purified by centrifugation (3000 rpm, 5 min) three times, with 150 μL 1×TE-Mg^{2}⁺-CA buffer (1×TE buffer with 10 mM CA, 10 mM $MgCl_2$).

1.3 Light-triggered, transcription machinery-guided, reconfiguration of compartmentalized

microdroplet condensates

For transcription machinery-guided reconfiguration of microcompartmentalized MD (Figure 1), MDs were prepared using oligonucleotides pA-Bs1. The transcription template T_{MG} tethered with the invading domain was formed by annealing T_{MG-1} and T_{MG-2} at the same concentration (2 μM). After treating the barcode-modified condensates with the invading transcription template T_{MG} (engineered to transcribe the MG RNA aptamer conjugate tethered to a barcode complementary sequence), the supernatant was removed, and the MDs were washed three times with 1×TE-Mg^{2+}-CA buffer according to the standard protocol. Subsequently, NTPs and T7 RNAP for transcription were added, diluted in 1x transcription buffer to 2 mM NTPs and 2 U/μL T7 RNAP (stored and used as per supplier instructions). Additionally, 2 μM malachite green (MG) was added to the transcription solution for aptamer imaging.

For the light-triggered, transcription-guided, dynamic reconfiguration of the compartmentalized MDs, Figure 2, three invading strands (T1, T2, S1) (each 6 μM) were mixed in 1×TE-Mg^{2+}-CA, followed by a snap-cooling protocol (95°C to 4°C in <2 min) to stabilize their secondary structure. Annealed invading strands (T1, T2, S1) mixture was stored at 4°C until further usage. The T1, T2, and S1 invading strands were subjected to the pA-Bs1 MDs at a final concentration of 2 μM of each invading strand, leading to a microcompartmentalized MDs in state J. After competitive invading of concentric three-compartment MDs, the excess invading strands were washed off three times with 1×TE-Mg^{2+}-CA buffer. The microcompartmentalized MD in state J were exposed to the UV irradiation (λ = 365 nm) for 3 min, with a specified power density of 4 mW/cm² at a distance

of 2 cm, and imaged by laser scanning confocal microscopy. The same UV condition can be used in the following experiments. Afterward, the NTPs and T7 RNAP for transcription were added, diluted in 1x transcription buffer to 2 mM NTPs and 2 U/μL T7 RNAP (stored and used as per supplier instructions). MD samples were recorded at different time points for confocal imaging.

1.4 Light-triggered, T7 RNAp-NTPs/RNase H-controlled, cyclic switchable, and transient reconfiguration of the microcompartmentalized MDs

MDs were prepared using oligonucleotide pA-Bs1 following a standardized protocol. Invading strands T3 duplex and hairpin S3 were diluted to 4 μM in 1×TE-$Mg^{2⁺}$-CA, respectively, followed by annealing processes: T3 underwent gradient thermal annealing (0.1°C/s ramp rate) to ensure precise duplex formation, while S3 was subjected to a snap-cooling protocol (95°C to 4°C in <2 min) to stabilize its secondary architecture. Annealed T3 and S3 were stored at 4°C until use. The purified MDs were subjected to T3 duplex and hairpin S3 to a final concentration of 2 μM. After the invasion process, the microcompartmentalized MDs were washed three times with 1×TE-$Mg^{2⁺}$-CA to remove the excess invading strands. The NTPs and T7 RNAP for transcription were added, diluted in 1x transcription buffer to a 2 mM NTP mix, and 2 U/μL T7 RNAP (stored and used as per supplier instructions). Samples were collected at different time points for confocal microscopy imaging. In the cycling experiment, the transcription buffer was replaced with RNase H hydrolase buffer (0.2 U/μL) in each cycle.

Additionally, in the experiment on dissipative transient reconfiguration of microcompartmentalized MDs, after the invading strand T3 duplex and hairpin S3 were

subjected to the same annealing, invasion, and washing procedures, RNase H diluted to the specified concentration was mixed with transcription components (including 2 mM NTPs mixture and 2 U/μL T7 RNA polymerase) and then added to the MDs. The samples were incubated at 37°C, and samples were Collected at different time points for confocal microscopy imaging.

1.5 Light-triggered T7 RNAp-NTPs/RNase H-dictated cyclic switchable, and transient reconfiguration of the microcompartmentalized MDs, accompanied by aggregation/deaggregation

MDs were prepared using oligonucleotide pA-Bs1 following a standardized protocol. Invading strands T4 duplex and hairpin S4 were diluted to 4 μM in1×TE-$Mg^{2⁺}$-CA, respectively, followed by a similar annealing procedure for the T3 duplex and hairpin S3. Annealed products were stored at 4°C until use. The washed MDs were mixed with T4 and S4 to a final 2 μM annealing concentration. After the invasion process, the microcompartmentalized MDs were washed three times with 1×TE-$Mg^{2⁺}$-CA to remove excess invading strands. For the cyclic aggregation/deaggregation experiments, we made use of the fact that the 15 um-sized condensates precipitate gravitationally from the aqueous buffer solution within a few minutes and in order to preserve the condensate structures during the transcription-induced aggregation or RNase H deaggregation that proceeded in substantially longer time, we eliminated their physical agitation and perform the reaction in a gravitationally settled configuration of the MDs. For the transcription-induced aggregation, the settled MDs were reacted in the transcription buffer (50 ul) that included T7 RNAp, 2 U/μl, and NTPs 2 mM for a time interval of 6 h (Under these condition samples, 1-2 μl

withdrawn from the system do not show any monomer MDs and only aggregated structures are observed). After imaging the aggregated assemblies, the transcription buffer was exchanged with the RNase H buffer and the hydrolytic digestion of the aggregates was performed for 3 h in the presence of RNase H, 0.2 U/μl (Under these conditions only separated MDs could be imaged). For cyclic aggregation/deaggregation experiments respective buffer and enzyme solutions on the deposited MDs were repeatedly exchanged.

Additionally, in the experiment on dissipative transient reconfiguration of microcompartmentalized MDs accompanied by aggregation/deaggregation, after the invading strand T4 duplex and hairpin S4 were subjected to the same annealing, invasion, and washing procedures, RNase H diluted to the specified concentration was mixed with transcription components (including 2 mM NTPs mixture and 2 U/μL T7 RNA polymerase) and then added to the MDs. The samples were incubated at 37°C, and samples were collected at different time points for confocal microscopy imaging. For control experiments with different concentration ratios of T7 RNAp/RNase H, the NTP concentration was fixed at 2 mM, and only the required concentrations of T7 RNAp and RNase H were varied.

1.6 The transcription machinery-guided intercommunication within microdroplet aggregates

Two different MDs were prepared following standard procedures using oligonucleotides pA-Bs1 (W)/pA-Bs1′ (V). W and V were mixed and hybridized, leading to the aggregated MDs W/V, followed by the invasion process using pre-annealed T1, T2, and S5 (2 μM for each strand). After competitive invasion of compartmentalized condensate

aggregates (W/V), the MDs were washed three times with 1×TE-Mg^{2+}-CA buffer to remove excess invading strands. The samples were exposed to 365 nm UV light for 3 minutes and then imaged using laser scanning confocal microscopy. NTPs and T7 RNA polymerase (T7 RNAP) for transcription were added, diluted in 1× transcription buffer to 2 mM NTPs and 2 U/μL T7 RNAP (stored and used as per the supplier's instructions). Samples were collected at different time points for confocal imaging.

It should be noted that in the control experiment, after preparing two different MDs (W' and V'), W' was invaded with pre-annealed T1 and T2, and V' was invaded with pre-annealed S5 separately before mixing. After the competitive invasion of the compartmentalized condensate aggregates (W'/V'), respectively, the MDs (W'/V') were each washed three times with 1×TE-Mg^{2+}-CA buffer to remove excess invading strands. Subsequently, W and V were mixed for the transcription experiment.

1.7 Statistical analysis of confocal fluorescence images, evolution of error-bars and sample volumes used record images

The experiment described in Figure 2 was reproduced by N=3 independent experiments while analyzing different 135×135 μm frames for each experiment, analyzing a total number of 54 condensate MDs. The average sizes and respective fluorescence intensities of the MD analyzed in the independent experiment demonstrated a ±10% deviation in size. For Figure 3 to Figure 7 the statistical analysis corresponding to the number of independent repeat experiments (N) number and sizes of analyzed frames and each experiment, number of MD analyzed in the experiment, sample sizes of the MD solution used for evaluation of the respective error bars are detailed in the respective Figure captions.

Figure 3: Error bars were extracted from N=3 experiments, for each experiment analyzing 5 large-area 135 μm×135 μm frames, total 56 Condensates. Sample volume of condensates in experiment 20 ul. Error bars represent standard deviations of $s = \sqrt{\frac{\sum_{i=1}^{n}\left(x_i - \bar{x}\right)^2}{n-1}}$ (mathematical equation)

Figure 4: Error bars were extracted from N=3 experiments, for each experiment analyzing 5 large-area 135 μm×135 μm frames, total 62 Condensates. Sample volume of condensates in experiment 20 μl. Error bars represent standard deviations of $s = \sqrt{\frac{\sum_{i=1}^{n}\left(x_i - \bar{x}\right)^2}{n-1}}$ (mathematical equation)

Figure 5: Error bars were extracted from N=3 experiments, for each experiment analyzing 10 large-area 135 μm×135 μm frames, total 52 Condensates. Sample volume of condensates in experiment 20 μl. Error bars represent standard deviations of $s = \sqrt{\frac{\sum_{i=1}^{n}\left(x_i - \bar{x}\right)^2}{n-1}}$ (mathematical equation)

Figure 6: Error bars were extracted from N=3 experiments, for each experiment analyzing 10 large-area 135 μm×135 μm frames, total 58 Condensates. Sample volume of condensates in experiment 20 μl. Error bars represent standard deviations of $s = \sqrt{\frac{\sum_{i=1}^{n}\left(x_i - \bar{x}\right)^2}{n-1}}$ (mathematical equation)

Figure S20: Error bars were extracted from N=3 experiments, for each experiment analyzing 5 large-area 135 μm×135 μm frames, total 56 Condensates. Sample volume of condensates in experiment 20 μl. Error bars represent standard deviations of $s = \sqrt{\frac{\sum_{i=1}^{n}\left(x_i - \bar{x}\right)^2}{n-1}}$ (mathematical equation)

1.9 Oligonucleotides

Table S1: DNA sequences used in MDs preparation experiments

Name	Sequence 5′→3′
pA-Bs1	AAAAAAAAAAAAAAAAAAAA-TTAGGATAGATATACGGGTTC
pA-Bs1′	AAAAAAAAAAAAAAAAAAAA-GAACCCGTATATCTATCCTAA

Note: The cyan sequence represents a polyadenylation fragment that forms a triplex with cyanuric acid. The pink, light green, and light blue sequences serve as barcode sequences for subsequent functional coding. All sequences were purchased from Hippo Biotechnology Co., Beijing, China, and further purified by high-performance liquid chromatography (HPLC) and verified by mass spectrometry (MS) analysis.

Table S2: DNA sequences used in dynamic reconfiguration of the T7RNAP catalytic domains experiment (including dynamic intercommunication, and information transfer between MDs experiment)

Name	Sequence 5′→3′
T_{MG-1}	**Cy3**-<br>GAACCCGTATATCTAGTCTAATACGACTCACTATAGGGATCCCGA<br>C<br>TGGCGAGAGCCAGGTAACGAATGGATCCATCTATCCTAA
T_{MG-2}	TTAGGATAGATGGATCCATTCGTTACCTGGCTCTCGCCAGTCGGG<br>ATCCCTATAGTGAGTCGTATTAGAC
**T1**	**Cy3**-CTTCACCAATCACACTCATGATCTC/**PC-<br>Linker**/CGTATTAGACTG<br>AATACGATGAGTGTGATTGGTGAAG/**PC-Linker**/TCTATCCTAA
**T2**	**Cy5**-TCAACATCAGTCTGATAAGCTACCCTATAGTGAGT/**PC-<br>Linker**/GAG<br>ATCCGTATTCAGTCTAATACGACTCACTATAGGGTAGCTTATCAGA<br>CTGATGTTGAGTATATCTATCCTAA
**S1**	**BHQ1**-TCAACATCAGTCTGATAAGCTA/**PC-<br>Linker**/ATCAGACTGATGTT

	GA/**6-FAM**/TGAACCCGTATATCTATCCTAA

Note: The bases highlighted in pink in the **T1**, **T2**, **S1**, and **T_{MG-1}** sequence were used to hybridize with the **Bs1** coding strand, the **T_{MG-2}** sequence is used to form a complete malachite green aptamer transcription template with **T_{MG-1}**. In particular, the T7 promoter in all templates is marked in yellow. All sequences were purchased from Hippo Biotechnology Co., Beijing, China, and further purified by high-performance liquid chromatography (HPLC) and verified by mass spectrometry (MS) analysis.

Table S3: T7RNAP/RNase H coupled catalytic cycle to switch DNA sequences used in dynamic reconfiguration experiments (including single MD reconfiguration and multiple MD aggregation/disaggregation).

Name	Sequence 5'→3'
**T3**	GTCTAATACGACTCACTATAGGGAACTACTTCTATCCTAAATC CGGTGAACCCGTATATCTATCCTAA-**Cy3**
**T3'**	CCGGATTTAGGATAGAAGTAGTTCCCTATAGTGAGTCGTATTAGA C
**S3**	**Cy5**- GGGAACTACTTCTATCCTAA/**PClinker**/CCGGATTTAGGATAGAAG TAGTTCCCGAACCCGTATATCTA
**T4**	TTAGGATAGAACATCAGTCTGATGTGCTACCCTATAGTGAGTCGT ATTAGACGTATATCTATCCTAA-**FAM**
**T4'**	GTCTAATACGACTCACTATAGGGTAGCACATCAGACTGATGTTCT ATCCTAA
**S4**	**Cy3**- ACATCAGTCTGATGTGCTACC/**PClinker**/ACATCAGACTGATGT/**Cy5**/ GAACCCGTATATCTATCCTAA
**S5**	**BHQ1**-TAGCTTATCAGACTG/**PC-Linker**/TCAACATCAGTCTGATAAGC TA/**6-FAM**/ TTAGGATAGATATACGGGTTC

Note: The pink bases in the **T3**, **T4**, **S3**, and **S4** sequences are used to hybridize with the Bs1 coding strand, the **T3'** and **T4'** sequences are used to form a complete T7 promoter transcription template with **T3** and **T4**, respectively, the photocleavable linker (PC-linker)

in the DNA sequence is used to photochemically release single-stranded DNA for DNA strand displacement reaction, and the bases highlighted in light green in **S5** sequence were used to hybridize with the **Bs1'** coding strand, the photocleavable linker (PC-linker) in DNA sequences are used to photochemically release single-stranded DNA for DNA strand displacement reactions and **BHQ-1** in the sequence was used to quench the **FAM** fluorescence. In particular, the T7 promoter in all templates is marked in yellow. All sequences were purchased from Hippo Biotechnology Co., Beijing, China, and further purified by high-performance liquid chromatography (HPLC) and verified by mass spectrometry (MS) analysis.

Table S4: Sequences for dynamic MD fusion experiment for a long time-interval in aggregated environment (control experiment for stability of the aggregated MDs W/V (Figure S22)).

Name	Sequence 5'→3'
**C**	**FAM**-ACTTGGGCATATTCTATCCTAA
**D**	**Cy5**- TCATGATCGTTATACGGGTTC

Note: The green highlighted bases in the sequence are used to hybridize with the Bs1 coding strand, and the red highlighted bases are used to hybridize with the Bs1' coding strand.

2. Supplementary Figures

Modified preparation of phase-separated MDs using PEG as a crowding agent.

Macromolecular crowding significantly influences reaction kinetics and the physical characteristics of cellular interiors[1]. PEG is commonly utilized as a macromolecular crowding agent to foster the aggregation of proteins and nucleic acids in vitro. From the

perspective of soft matter physics, PEG predominantly modulates the "nucleation and growth" stages of bio-macromolecular aggregation processes.[2-4] In this consideration, we applied PEG as a crowding agent to facilitate the phase-separated formation of CA/pA DNA MDs.

Figure S1 depicts schematically the self-assembly of phase-separated triplex-stabilized cyanuric acid (CA)/polyadenine (pA) crosslinked DNA microdroplets (MDs). Annealing of polyadenine strands tethered to barcode nucleic strands (pA-Bs1) leads, in the presence of a delicate concentration balance of Mg^{2+} ions, CA, pA-Bs1, and an optimized PEG-800 (Figure S1, Panel I), to the formation of pA-Bs DNA condensates in a continuous aqueous environment. The phase separation mechanism of the condensed DNA MDs is schematically depicted in Panel II and involves the primary formation of rosette CA-stabilized pA-triplexes, followed by their aggregation into fibers and their crosslinked coagulation into phase-separated MDs. The phase diagram shows the broadened threshold concentrations and concentration ranges of the constituents leading to the facilitated phase-separated MDs, in the presence of PEG-800. The confocal microscopy images of the CA/pA-Bs1 MDs are shown in Figure 1(C), Panel I. The yield of the MDs corresponded to ca. 90% and the average size of the MDs corresponded to ca. 15 μm. The resulting pA-Bs1 coagulated MDs are functionalized with the barcode Bs strand, Bs1 composed of a pre-engineered sequence that can hybridize with auxiliary "invading" strands composed of variable lengths of complementary base sequences, resulting in concentric compartmentalized MDs, which was well explained in our previous study.

We incorporated PEG as a crowding agent to facilitate the phase-separated MDs, by exploring the effects of PEG molecular weight (Figure S2) and PEG-800 concentration (Figure S3) on the morphology of the resulting MDs. Furthermore, we optimized the concentrations of key components, including DNA pA-Bs1 (equivalent to 20x the concentration of base A, Figure S3), Mg^{2+}-ions (Figure S4), and CA (Figure S5). Our findings revealed that well-defined DNA MDs condensates with moderate size and well-defined spherical structure (average particle size of 15 ± 4.3 μm, as determined by ImageJ analysis) could be obtained when the pA-Bs1 was set at 10 μM (equivalent to 200 μM of base A). Notably, the influence of Mg^{2+} ions on DNA coagulation is significantly more pronounced than that of CA. Specifically, within the range of 10-100 mM Mg^{2+} concentration, phase-separated MDs of clear structure, moderate size, and good dispersion could be obtained. While at Mg^{2+} concentrations below 5 mM, DNA fails to coagulate effectively, resulting in amorphous aggregates. Conversely, when Mg^{2+} concentration exceeds 100 mM, initially dispersed MDs aggregate into clusters. For CA, theoretically, the molar ratio of CA to adenine base in the system should exceed 3:1.[5] Optimized microdroplet condensates with good morphology and high yield can be obtained with 6 mM CA.

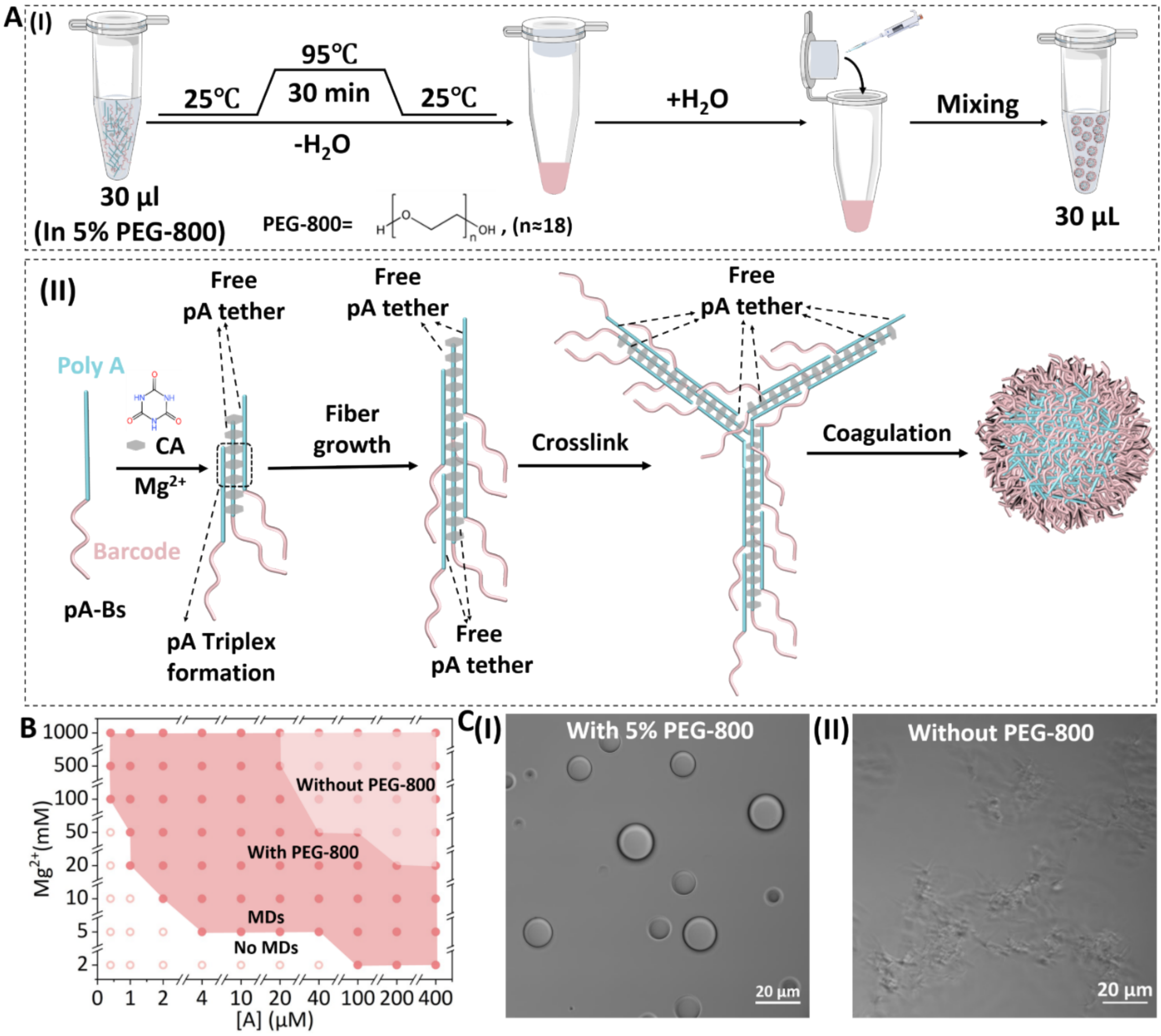

Figure S1. (A) Schematic preparation of phase separated DNA MDs composed of pA-Bs-tethered strand and CA in the presence of PEG-800 (Panel I), through the assembly of pA-Bs/CA triplex structures, their oligomerization into fibril intermediates that aggregate into the barcode Bs-tethered pA/CA MDs. (B) Phase diagram corresponding to the concentrations of the constituent generating the MDs in the presence of PEG-800, or in the absence of PEG-800. (C) Confocal brightfield images with (Panel I) and without (Panel II) 5% of PEG-800, under the conditions of CA concentration at 6 mM, Mg^{2}⁺ concentration at 50 mM, DNA concentration at 10 μM pA-Bs1 (Scale bar = 20 μm).

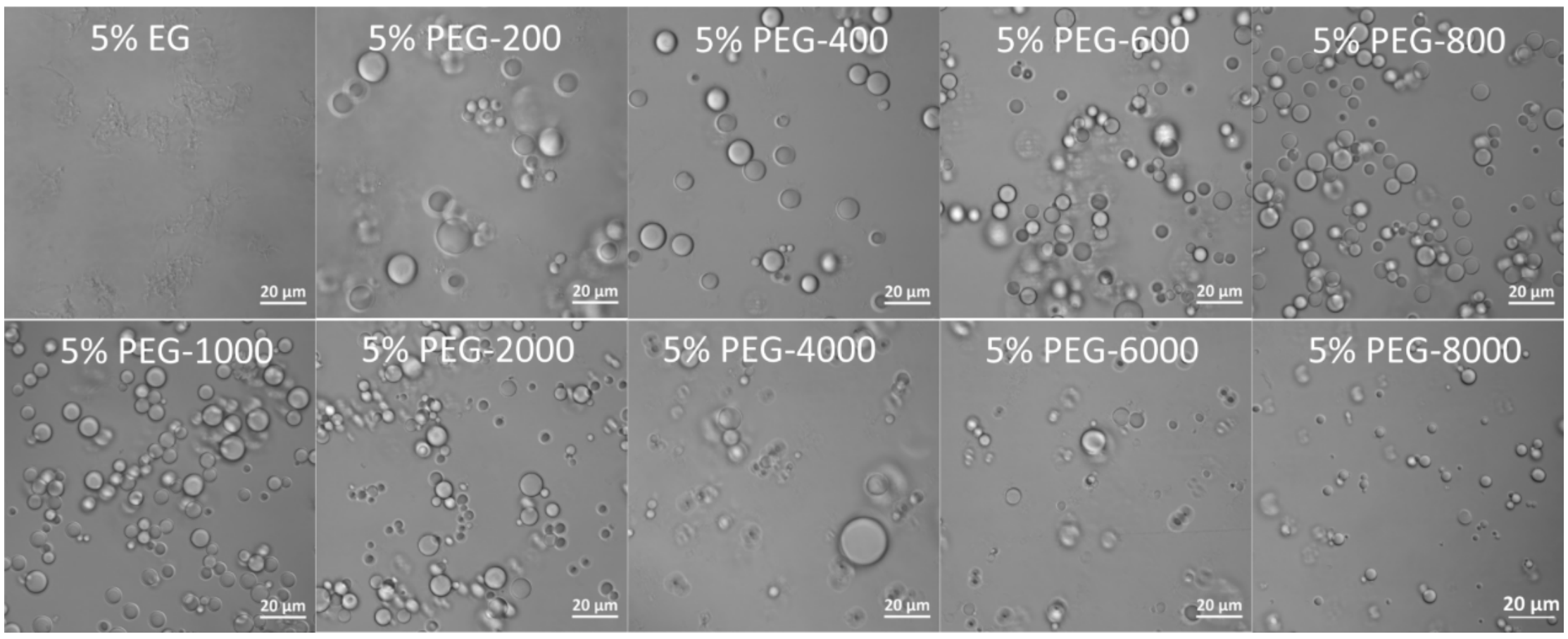

Figure S2. Confocal microscopy bright field images corresponding to effect of 5% PEG with different degree of polymerization on the morphologies of resulting MDs, using10 μM pA-Bs1, 50 mM Mg^{2+}-ion, and 6 mM CA. The molecular weight of PEG varies from 62 (monomer, ethylene glycol, EG) to 8000 (PEG 8000) (Scale bar = 20 μm).

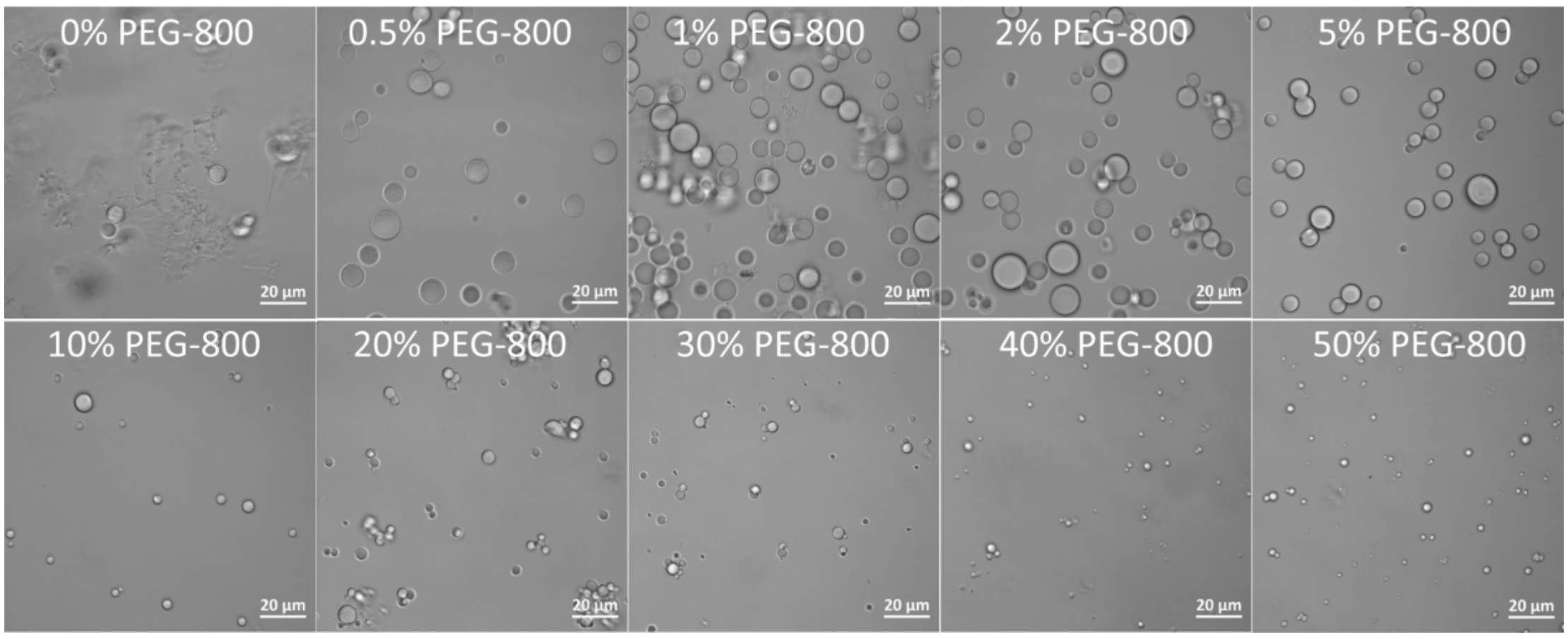

Figure S3. Confocal microscopy bright field images corresponding to effects of concentrations (ratio of w/w, from 0% to 50%) of PEG-800 on the morphologies of resulting MDs, in the presence of 10 μM pA-Bs1, 50 mM Mg^{2+}-ion and 6 mM CA (Scale bar = 20 μm).

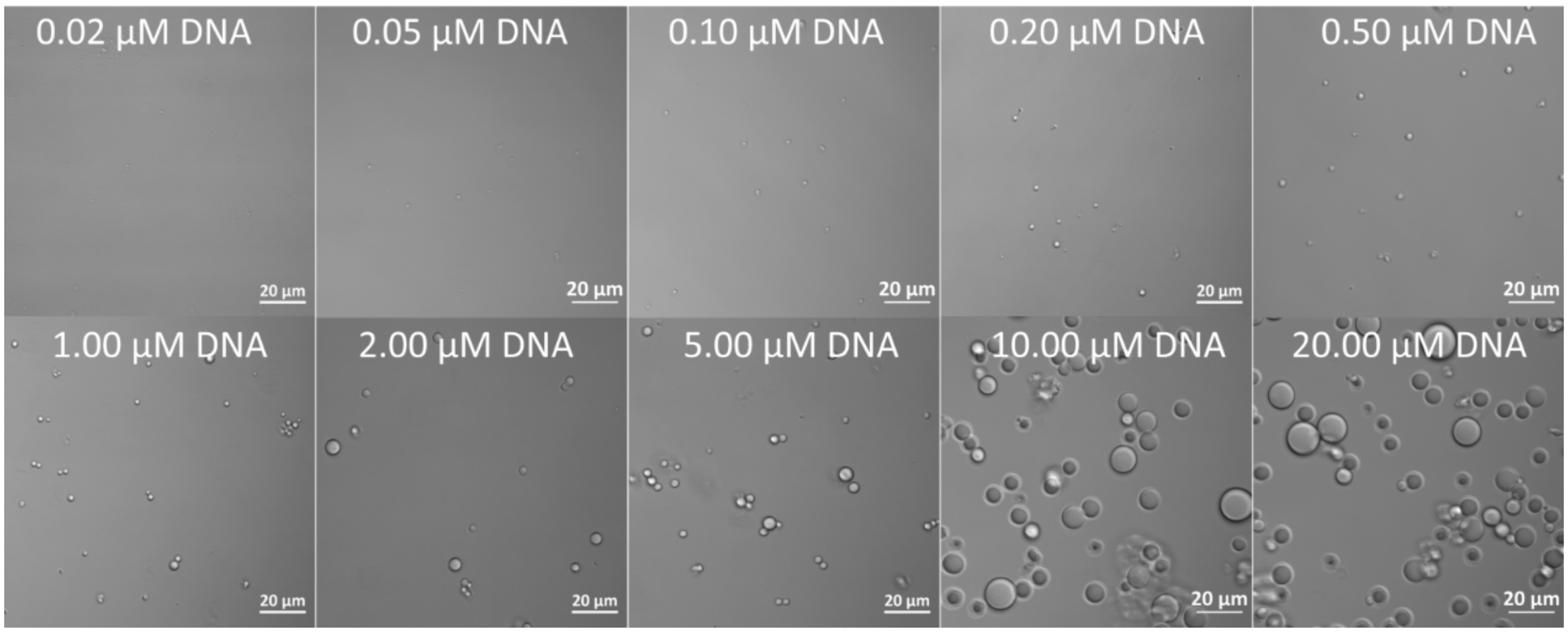

Figure S4. Confocal microscopy bright field images corresponding to effect of pA-Bs1 concentrations (from 0.02 μM to 20 μM) on the morphologies of resulting MDs, in the presence of 50 mM Mg^{2+}-ion, 5% PEG-800 and 6 mM CA (Scale bar = 20 μm).

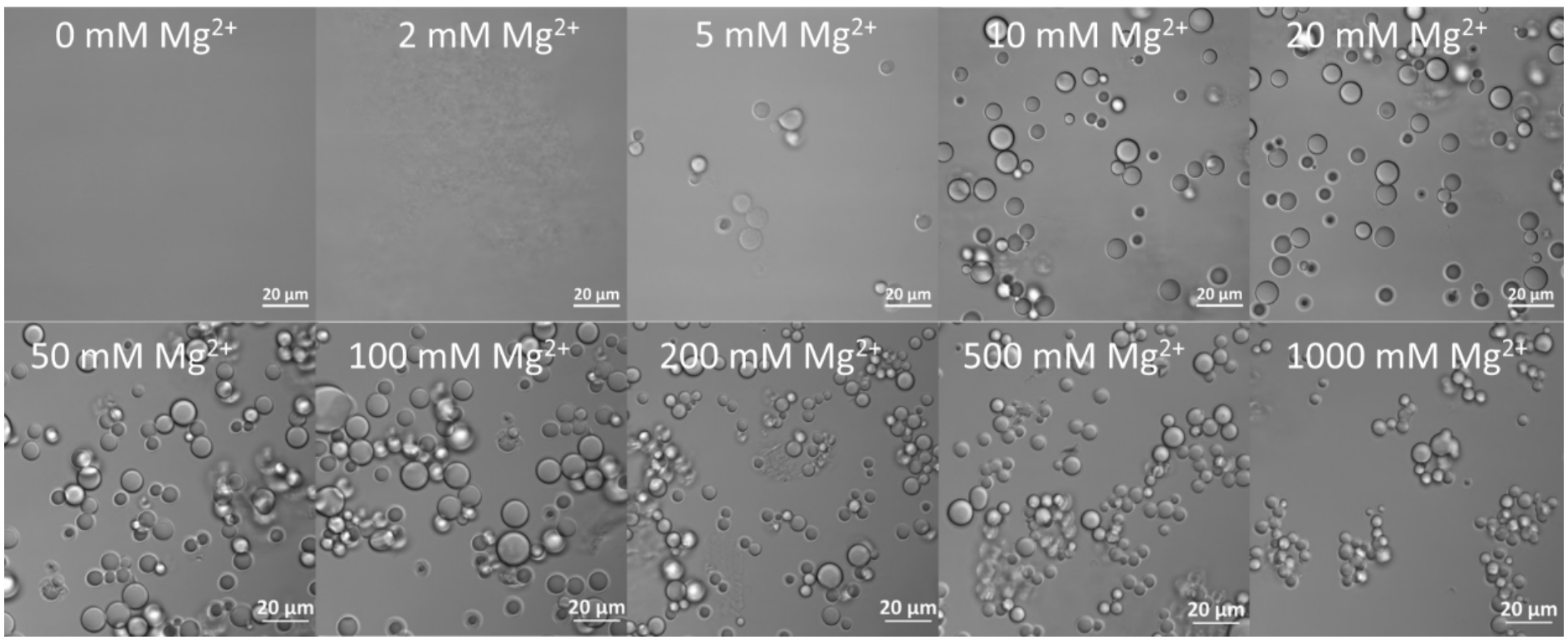

Figure S5. Confocal microscopy bright field images corresponding to effect of Mg^{2+}-ion concentrations (0 mM to 1 M) on the morphologies of resulting MDs, in the presence of 10 μM pA-Bs1, 6 mM CA and 5% PEG-800 (Scale bar = 20 μm).

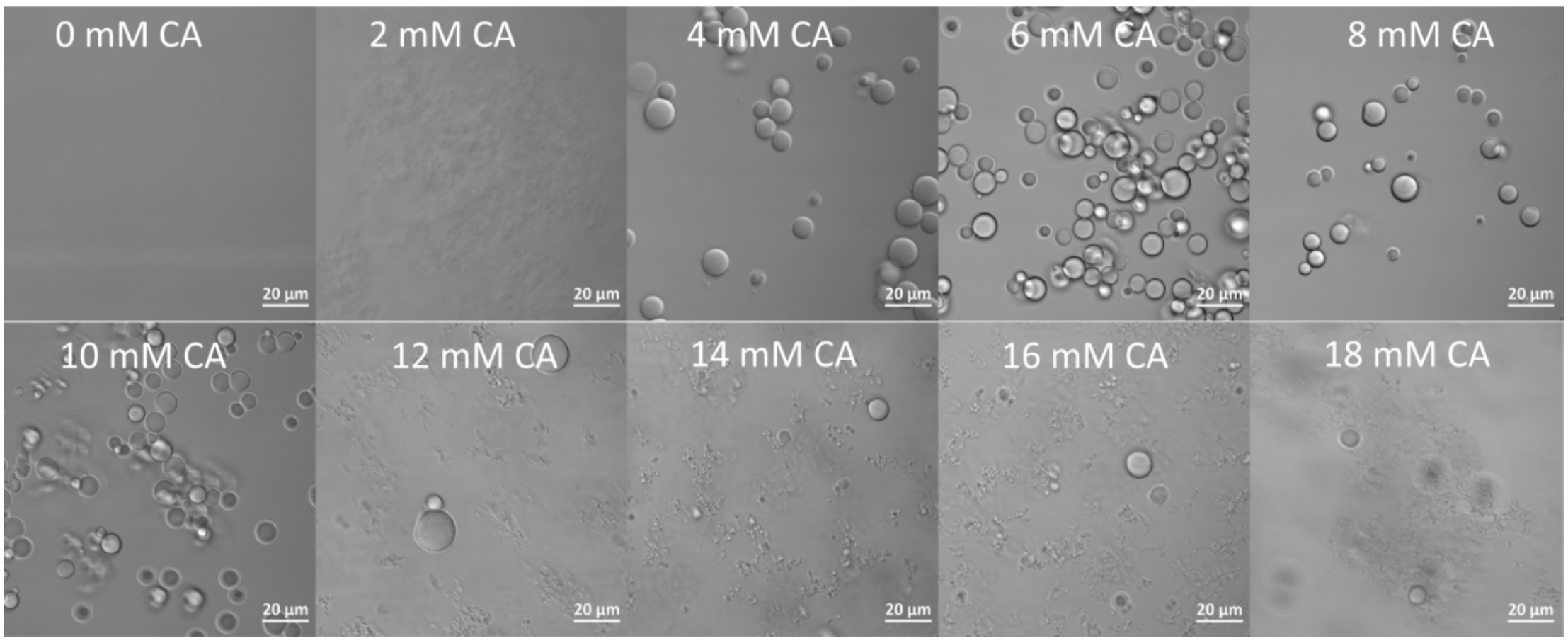

Figure S6. Confocal microscopy bright field images corresponding to effects of CA concentrations (0 mM to 18 mM) on the morphologies of resulting MDs, in the presence of 10 μM pA-Bs1, 50 mM Mg^{2+}-ion and 5% PEG-800 (Scale bar = 20 μm).

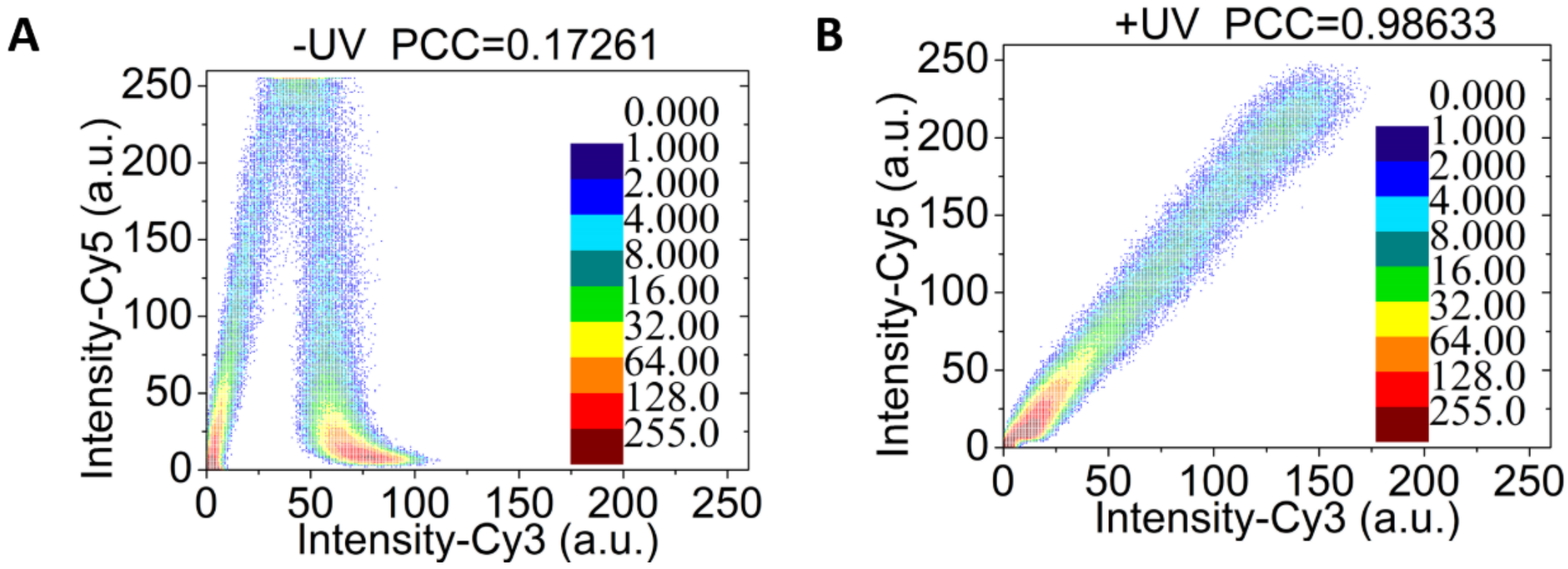

Figure S7. The Pearson's correlation coefficient (PCC) calculated for the Cy3 and Cy5 channel images of microcompartmnetalized MDs in state J (A) and in state K (B), as shown in Figure 2(B).

As shown in Figure S7, the person's correlation coefficient (PCC) values corresponding to Cy3 and Cy5 channels in microcompartmentalzied MDs (Figure 2(B)) in state J and state K, were evaluated respectively. Obviously, the microcompartmentalized MDs in state J before photochemical uncaging, showed a low PCC value of 0.17261, demonstrating a low correlation between Cy3 and Cy5 signals associated with compartmentalized MDs in state J, consistent with the separated blue Cy3 fluorescent core and red Cy5 middle compartment. After photochemical uncaging, the reconfigured microcompartmentalized MDs in state K showed a high PCC value of 0.98633, indicating a very strong correlation between Cy3 and Cy5 signals associated with the MDs in state K, consistent with the translocation of Cy3-modified T1' to red compartment comprising of Cy5-modified T2'-Bs1 and the formation of merged violet Cy3/Cy5 colocalized compartment composed of T1'-T2'-Bs1.

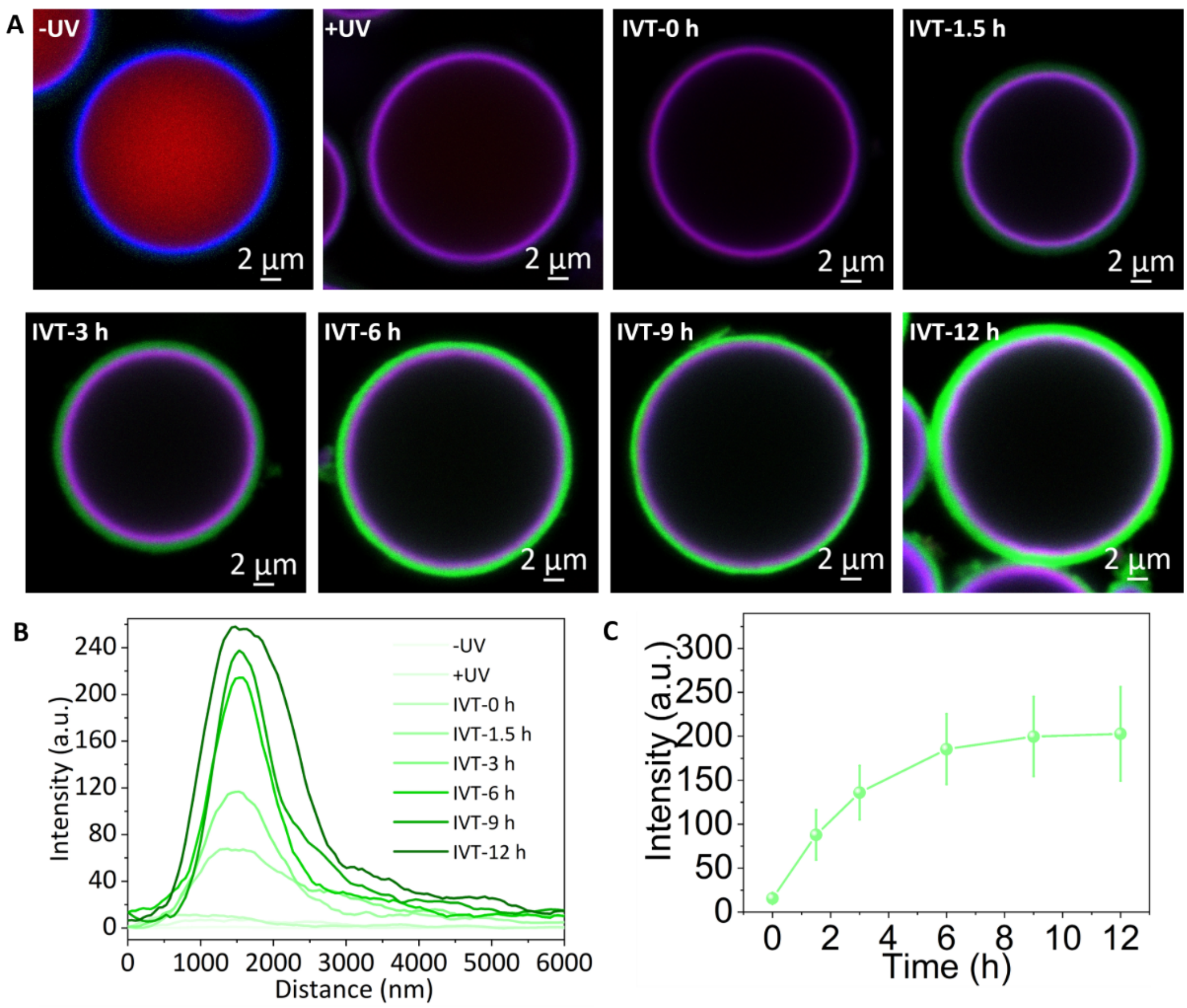

Figure S8. (A) The merged (green (FAM), blue (Cy3), and red (Cy5)) confocal fluorescence microscopy images corresponding to a single compartmentalized MD in state J before UV irradiation, in state K after photochemical UV uncaging, and temporal transitions from state K to state L after activation of transcription machinery (Scale bar = 2 μm). (B) The temporal fluorescence profiles or (C) Temporal intensity fluorescence changes corresponding to the integrated green fluorescence of the transcription product R1-S1'-Bs1, extracted from Fig.S8(A).

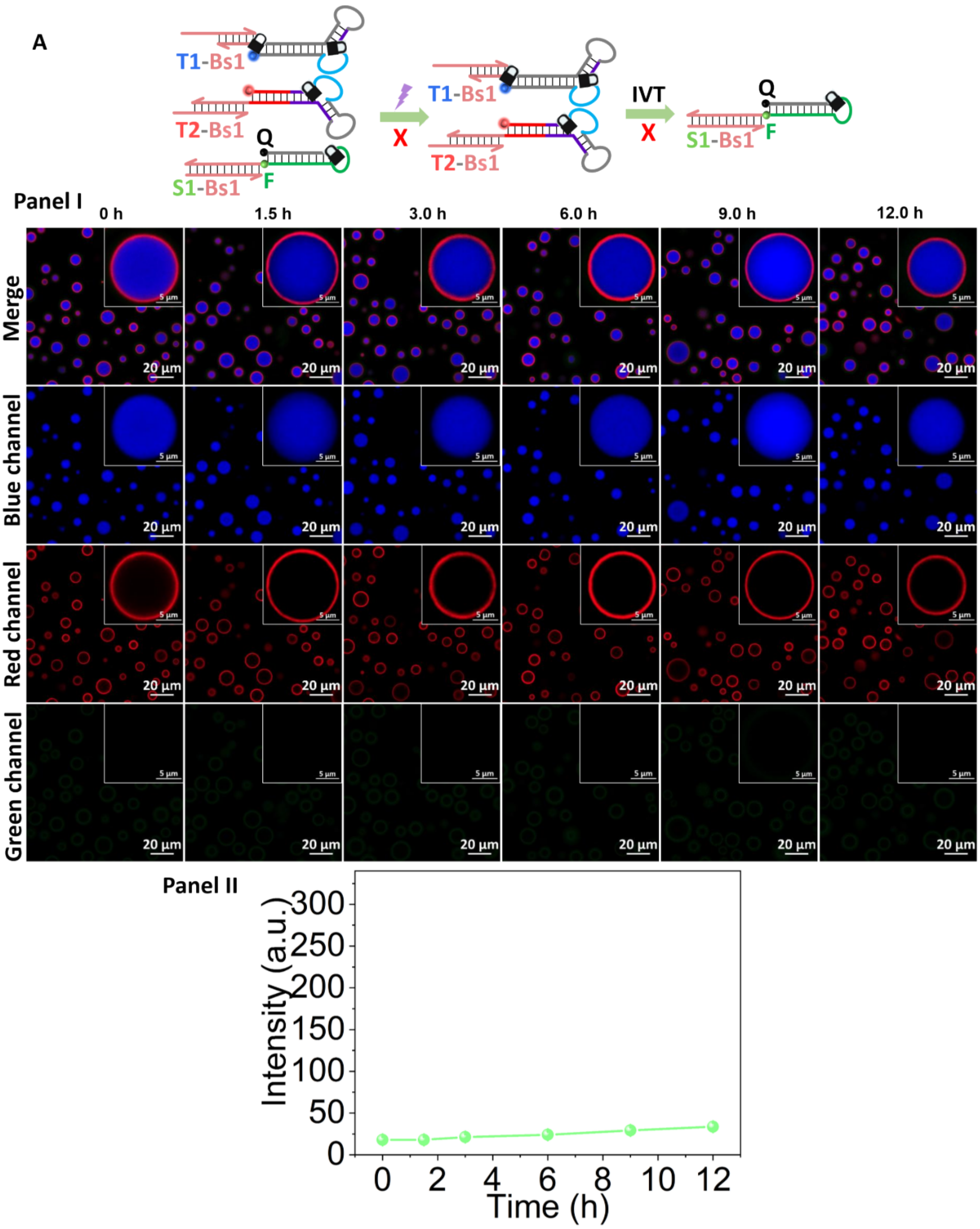

Figure S9. (A) Schematic control experiment of subjecting compartmentalized MDs in state J (Figure 2(A)) to T7 RNAp/NTPs without UV photodeprotection of the constituents associated with the MDs. Panel I is the time-dependent confocal fluorescence images of the MDs without the UV irradiation at different time intervals. Panel II is the temporal intensity fluorescence changes corresponding to the integrated green fluorescence of the transcription product R1-S1'-Bs1, extracted from Panel I.

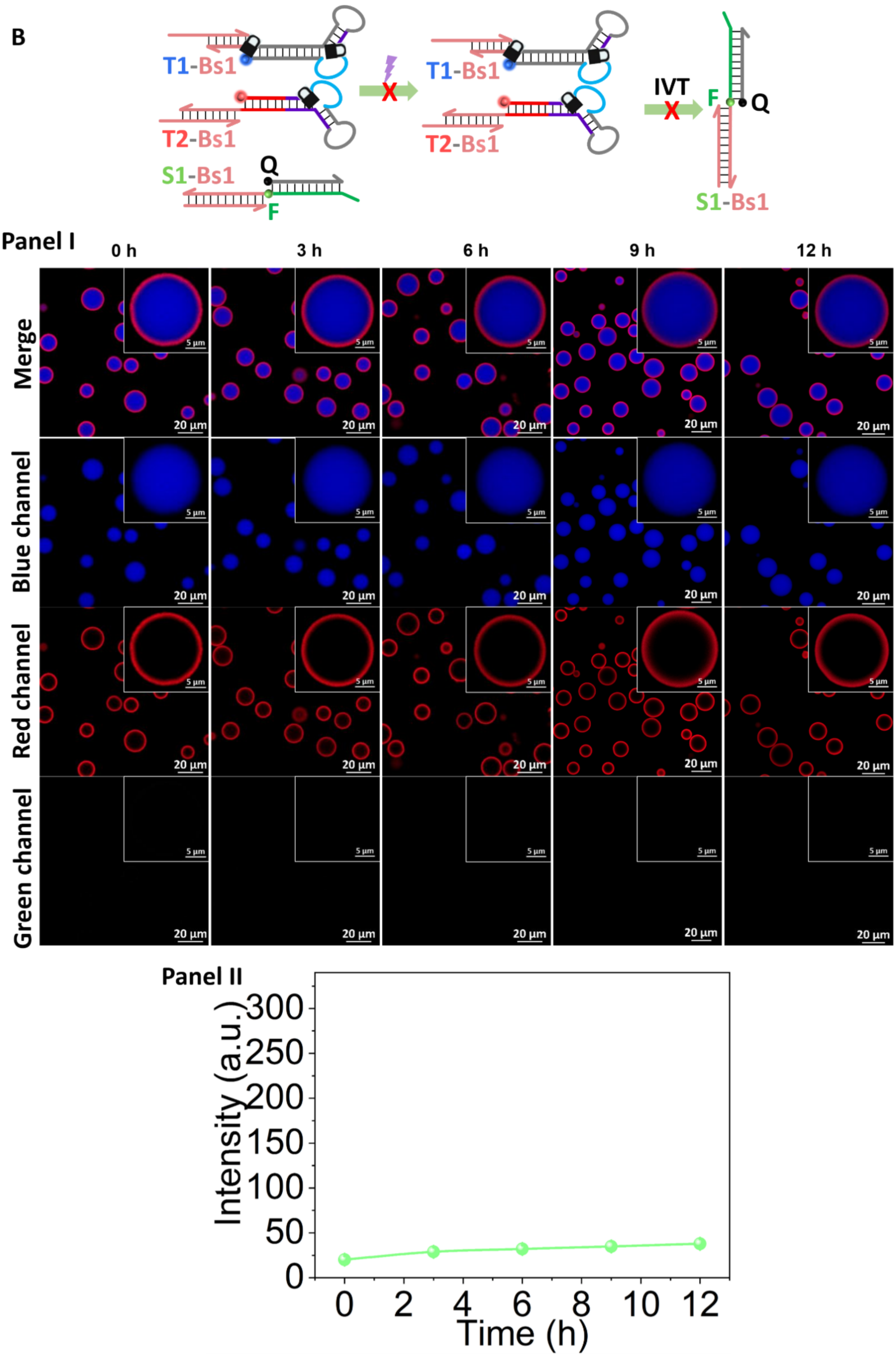

Figure S9. (B)Schematic control experiment of subjecting T1/T2/S1'-invaded compartmentalized MDs to T7 RNAp/NTPs without UV photodeprotection of the

constituents associated with the MDs. Panel I is the time-dependent confocal fluorescence images of the condensates with the non-dormant F/Q modified S1-Bs1 substrate and non-UV irradiation at different time intervals. Panel II is the temporal intensity fluorescence changes corresponding to the integrated green fluorescence of the transcription product R1-S1'-Bs1, extracted from Panel I.

Several control experiments supporting the light-triggered reconfiguration of the MDs microcompartmentalized coacervates as outlined in Figure 2 were performed:

The results presented in Figure S9(A), confirm that the light-triggered deprotection of the strands T1, T2, and S1-Bs1 as an essential step to induce the transcription-guided reconfiguration of the microcompartment. In this experiment the MDs are invaded with the strands T1, T2 and S1-Bs1 and resulting coacervates are subjected to the IVT process. Evidently, no transcription-induce compartment reconfiguration is observed (Panel II), confirming that the primary photoresponsive deprotection is essential to reconfigure the compartment by the transcription process.

In the second control experiment, Figure S9(B), the coacervates are subjected to two photoresponsive caged strands T1 and T2 and to a non-dormant substrate F/Q-modified S1-Bs1 substrate. Subjecting the system to IVT does not lead to any reconfiguration of the microcompartment of the coacervates even after a prolonged time interval of 12 h, Panel II. These results imply that any, possible, leakable formation of T1'-T2' incomplete transcription template generating R1 product, displacing the non-dormant substrate can be excluded.

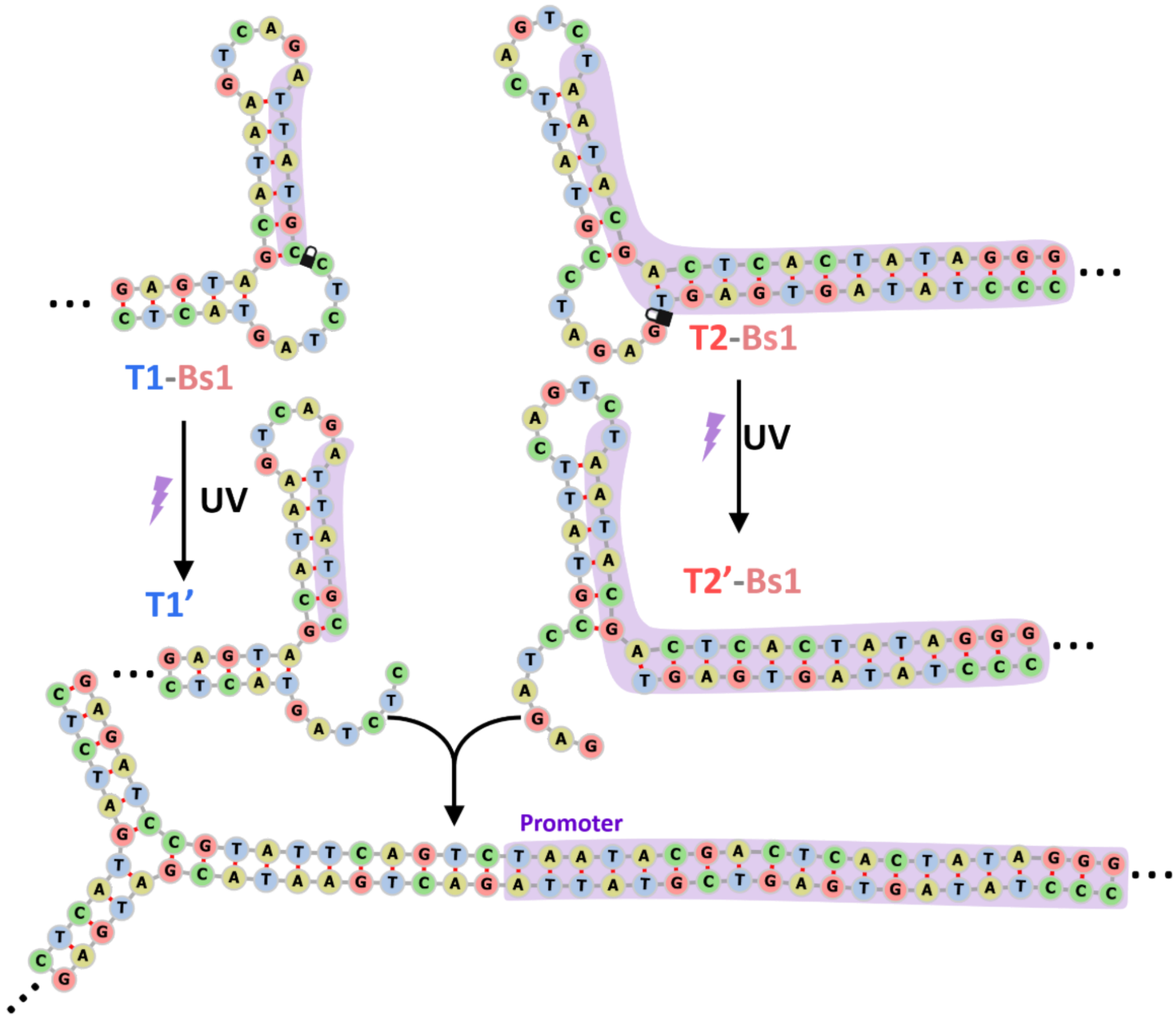

Figure S10. Detailed schematic of photoactivation-driven assembly of the transcription template.

Permeation of T7 RNAp and RNase H in the condensates.

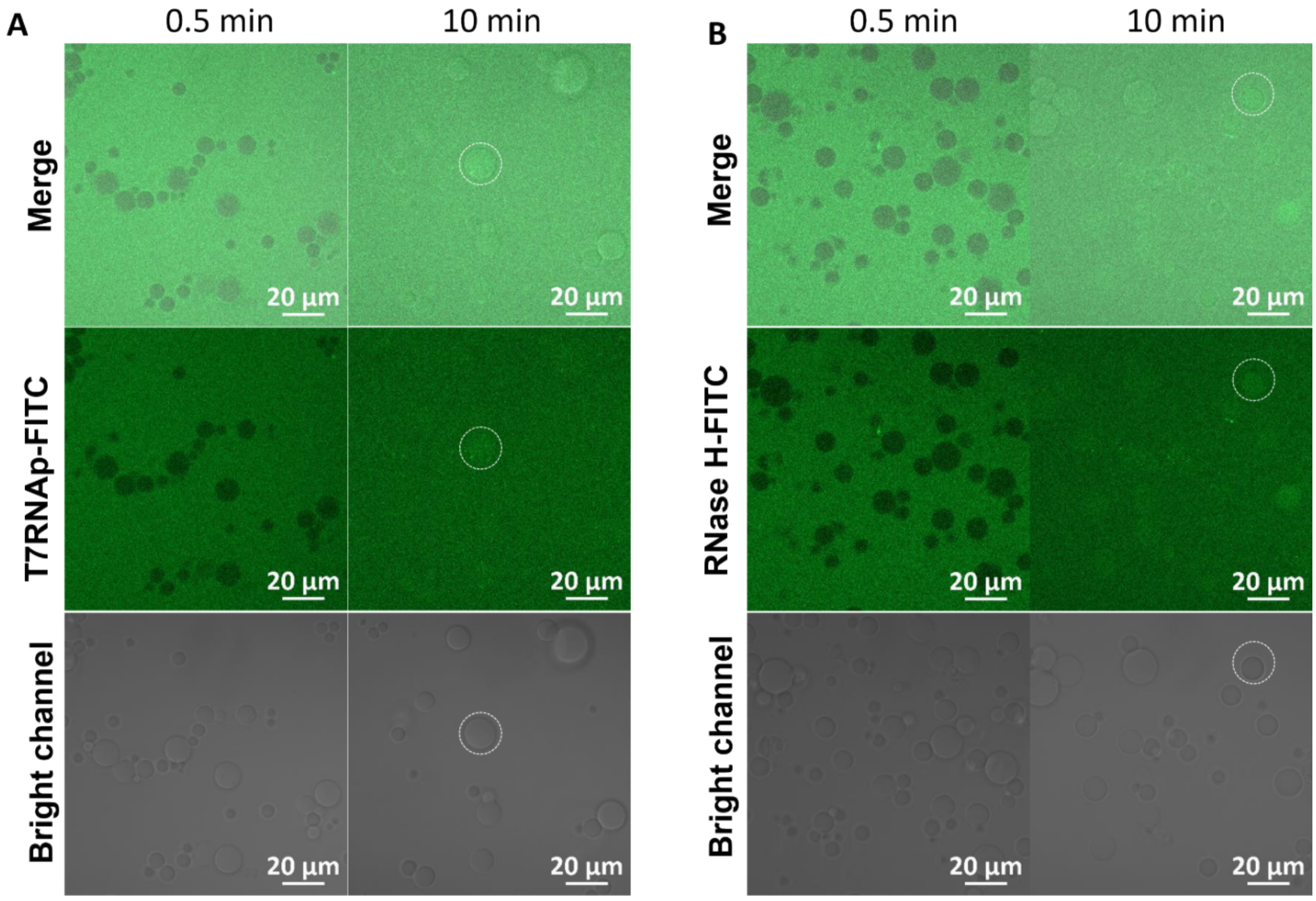

Figure S11. (A) Temporal confocal fluorescence images corresponding to subjecting MDs to FITC-modifed T7 RNAp, demonstrating permeability of the T7 RNAp into the MDs within 10 min. (B) Temporal confocal fluorescence images corresponding to subjecting MDs to FITC-modifed RNase H demonstrating permeability of the RNase H into the MDs within 10 min.

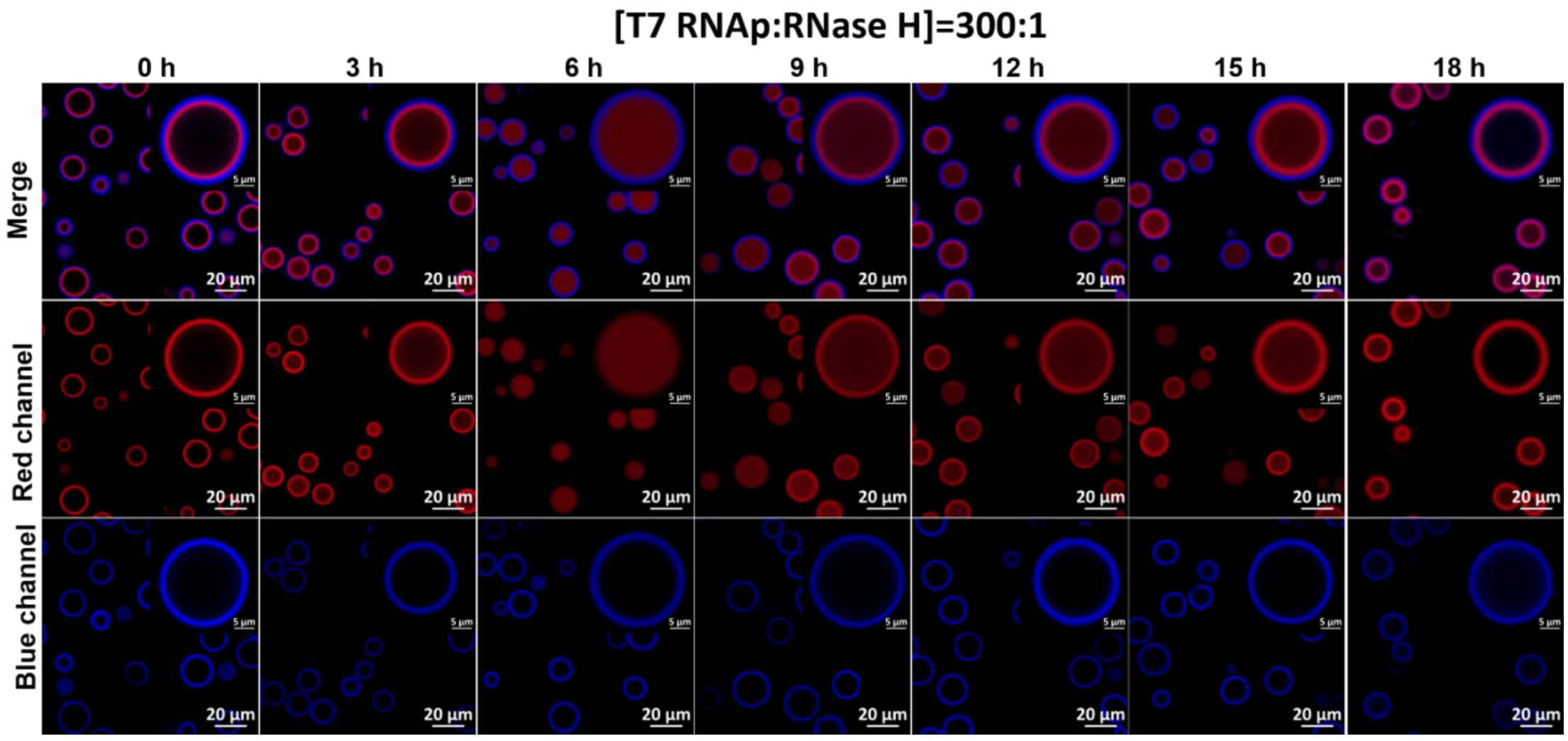

Figure S12. Confocal fluorescence microscopy images corresponding to the transient, dissipative, and temporal reconfiguration of microcompartmentalized MDs from state M to state N, and back to state M, driven by T7 RNAp/RNase H, at a T7 RNAp concentration of 2 U/μL and RNase H concentration of 0.0067 U/μL. The derived temporal integrated red fluorescence changes in the core compartment of the MDs corresponds to Figure 4(C), curve (ii) (scale bar = 20 μm, inset scale bar = 5 μm).

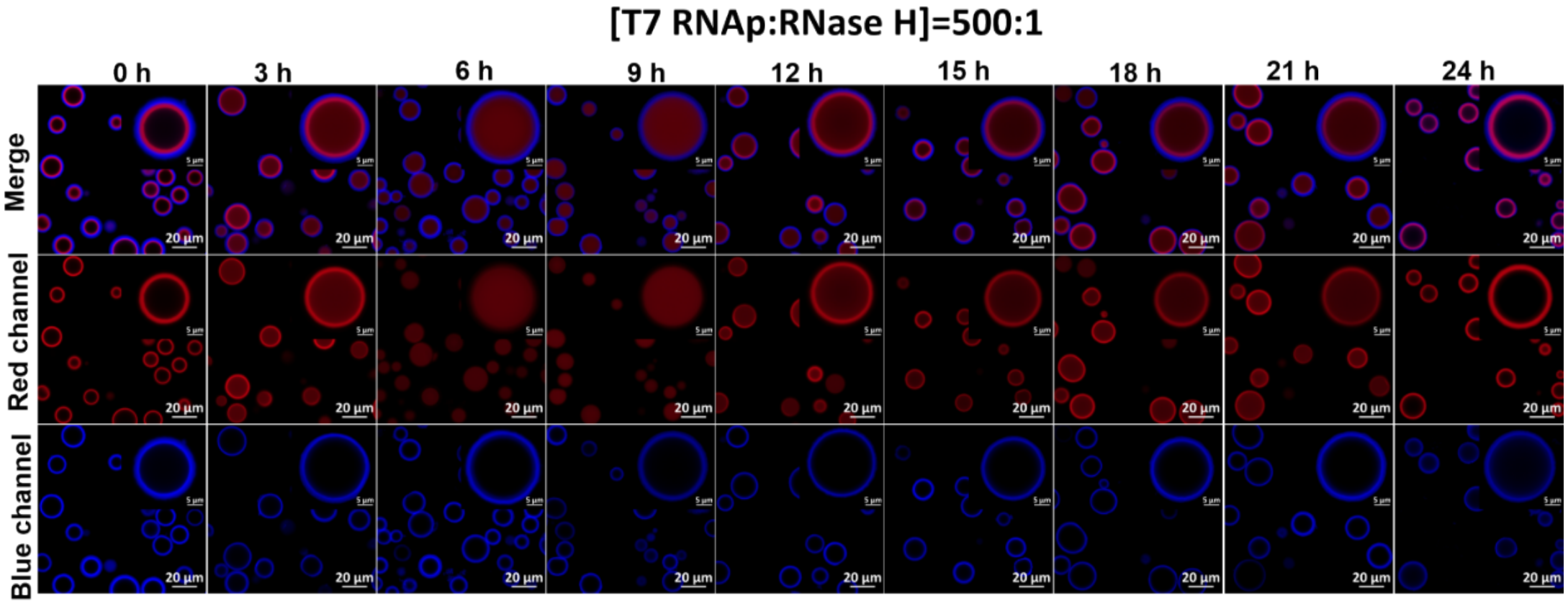

Figure S13. Confocal fluorescence microscopy images corresponding to the transient, dissipative, and temporal reconfiguration of microcompartmentalized MDs from state M to state N, and back to state M, driven by T7 RNAp/ RNase H, at a T7 RNAp concentration of 2 U/μL and RNase H concentration of 0.004 U/μL. The derived temporal integrated red fluorescence changes in the core compartment of the MDs corresponds to Figure 4(C), curve (iii) (scale bar = 20 μm, inset scale bar = 5 μm).

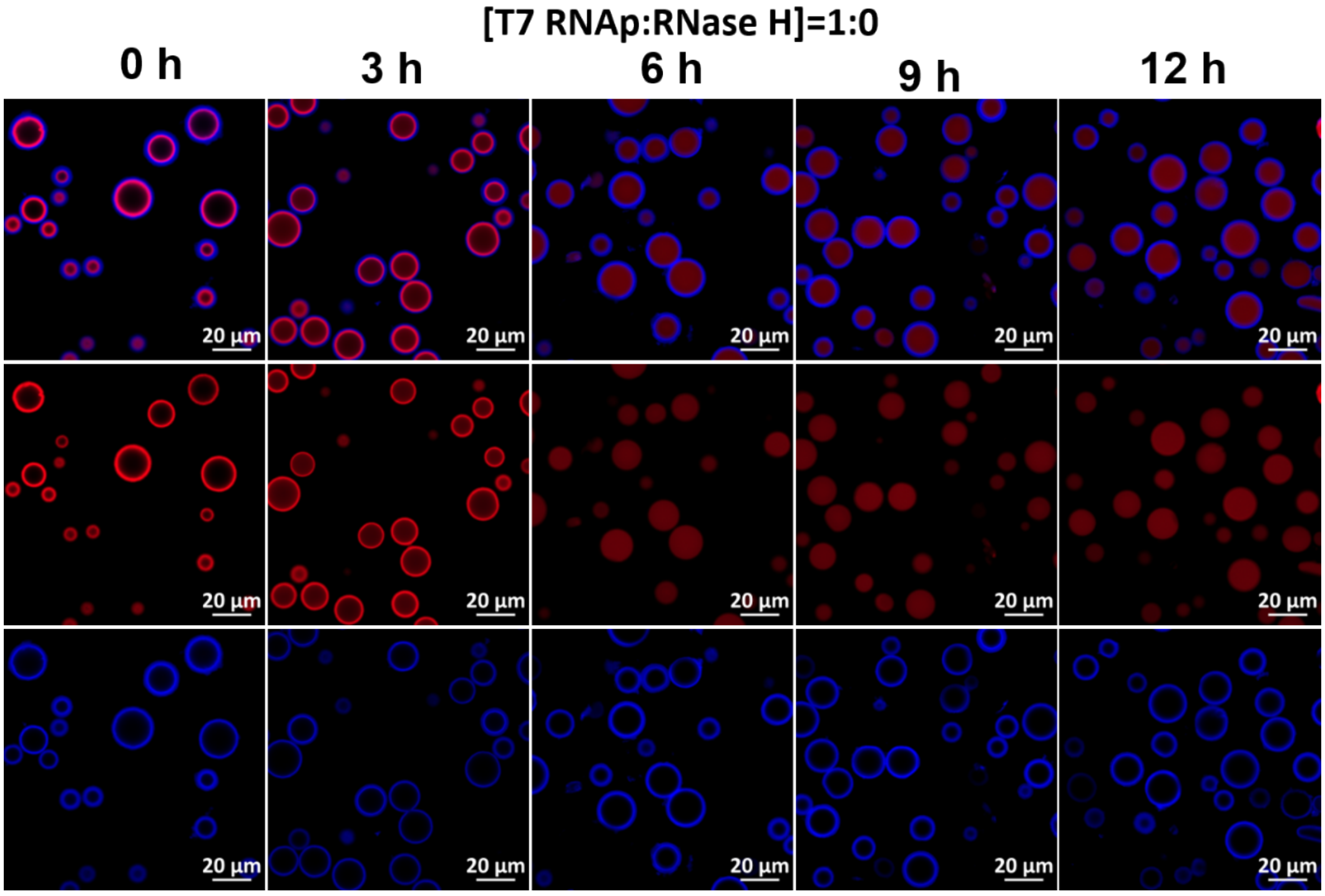

Figure S14. Confocal fluorescence microscopy images corresponding to control system of reconfiguration of microcompartmentalized MDs from state M to state N, in the presence of only 2 U/μL T7 RNAp without RNase H. The derived temporal integrated red fluorescence changes in the core compartment of the MDs corresponds to Figure 4(D), curve (i) (scale bar = 20 μm).

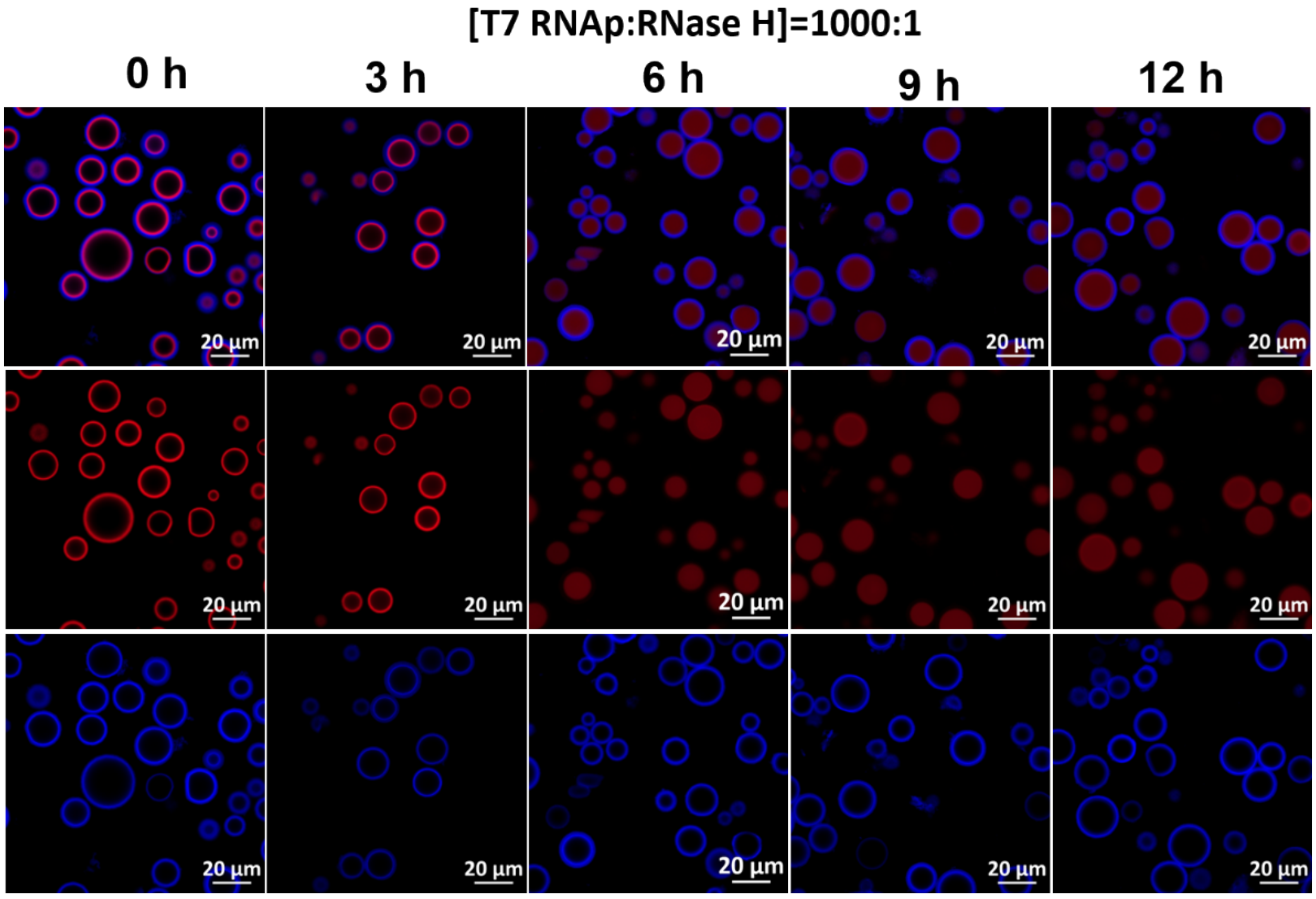

Figure S15. Confocal fluorescence microscopy images corresponding to control system of reconfiguration of microcompartmentalized MDs from state M to state N, in the presence of 2 U/μL T7 RNAp, and a low concentration of RNase H, 0.002 U/μL. The derived temporal integrated red fluorescence changes in the core compartment of the MDs corresponds to Figure 4(D), curve (ii) (scale bar = 20 μm).

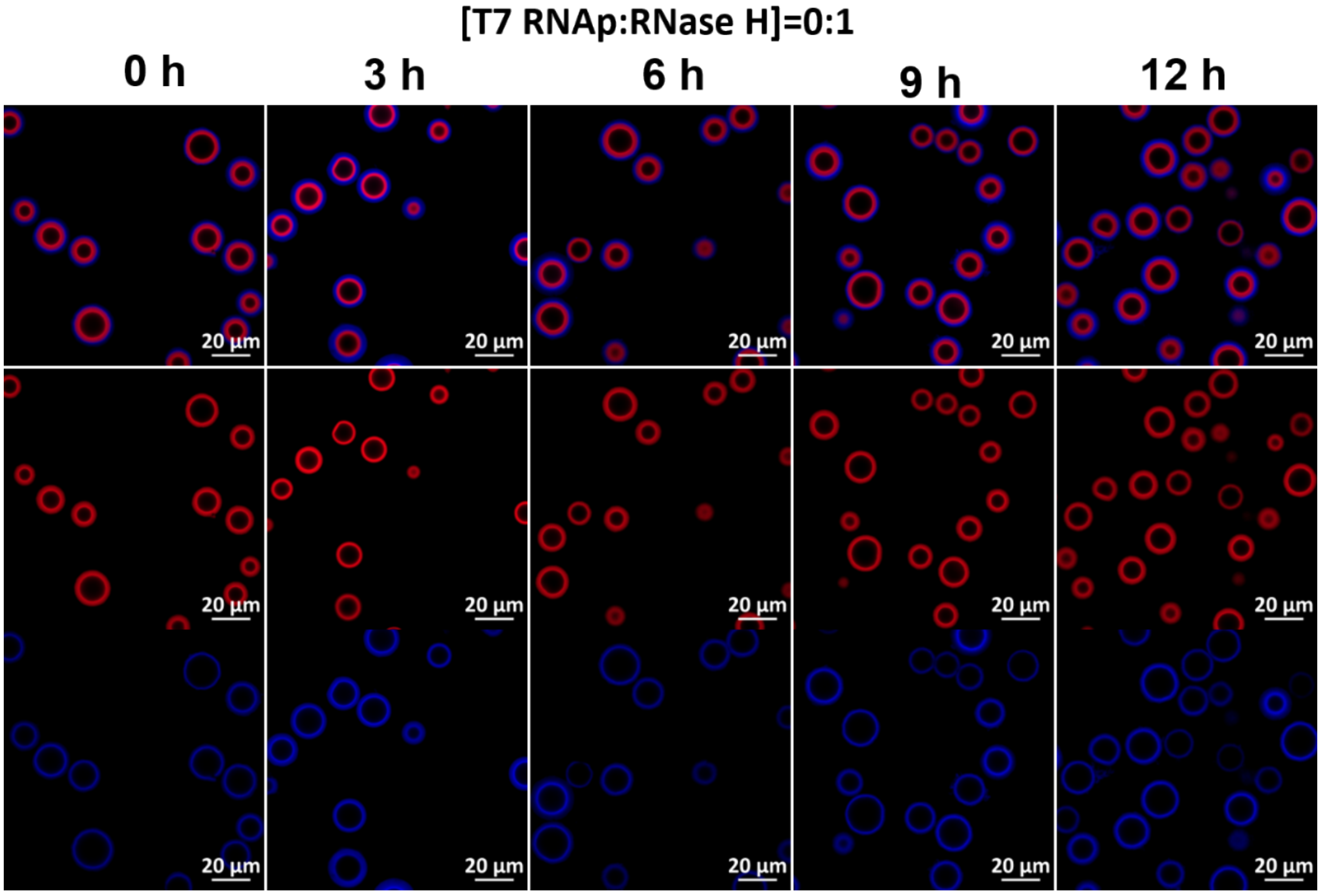

Figure S16. Confocal fluorescence microscopy images corresponding to control system of non-reconfigured microcompartmentalized MDs in state M, in the presence of only 0.2 U/μL RNase H without T7 RNAp. The derived temporal integrated red fluorescence changes in the core compartment of the MDs corresponds to Figure 4(D), curve (iii) (scale bar = 20 μm).

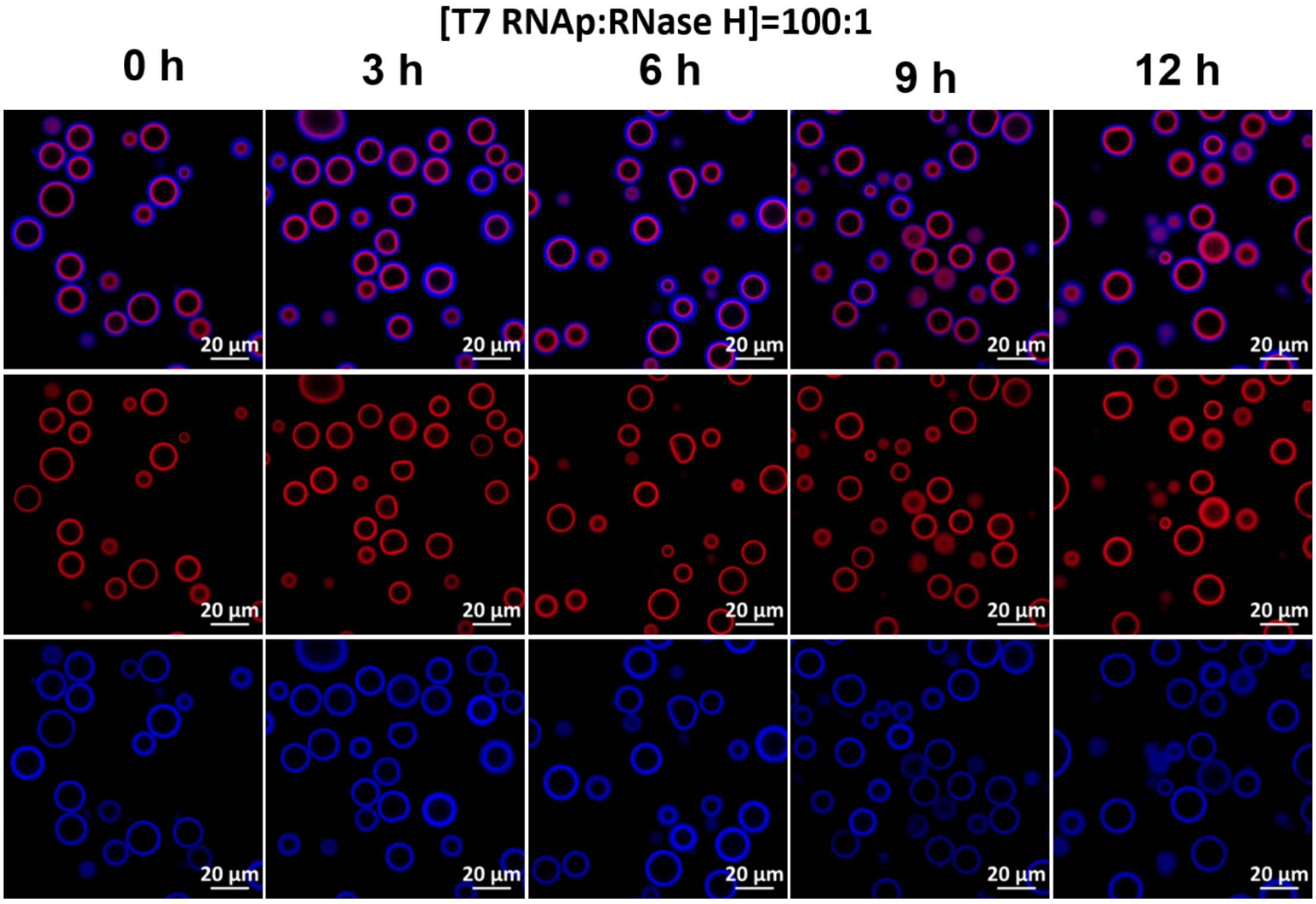

Figure S17. Confocal fluorescence microscopy images corresponding to control system of non-reconfigured microcompartmentalized MDs in state M, in the presence of only 0.02 U/μL RNase H without T7 RNAp and a low concentration of T7 RNAp, 2 U/μL. The derived temporal integrated red fluorescence changes in the core compartment of the MDs corresponds to Figure 4(D), curve (iv) (scale bar = 20 μm).

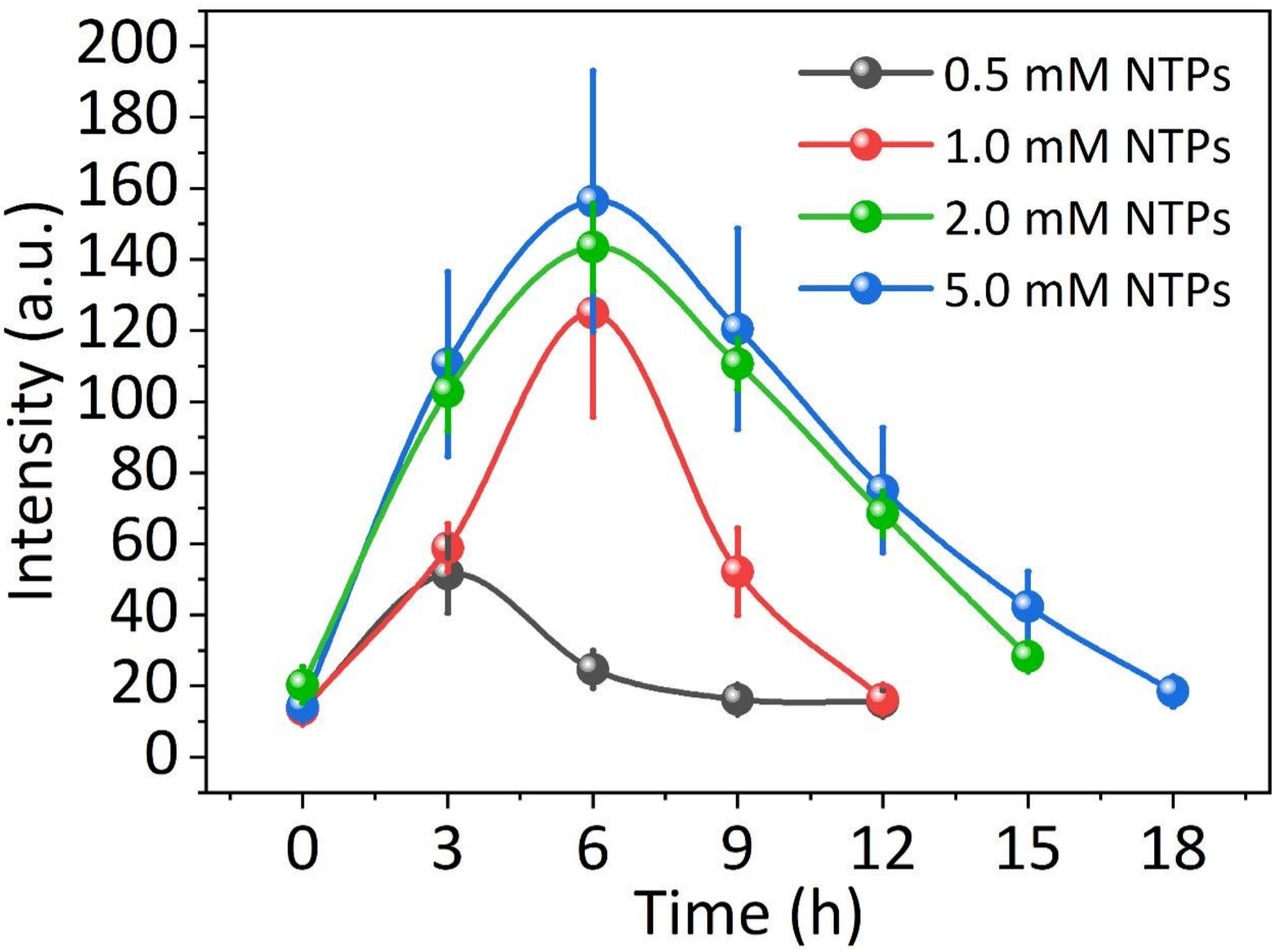

Figure S18. Transient fluorescence intensities changes of the red fluorophore (Cy5) upon dynamic transient transition of compartmentalized MDs between State M to State N and back in the presence of different NTPs concentrations. (T7 RNAp 2 U/μl and RNase H 0.01 U/μl)

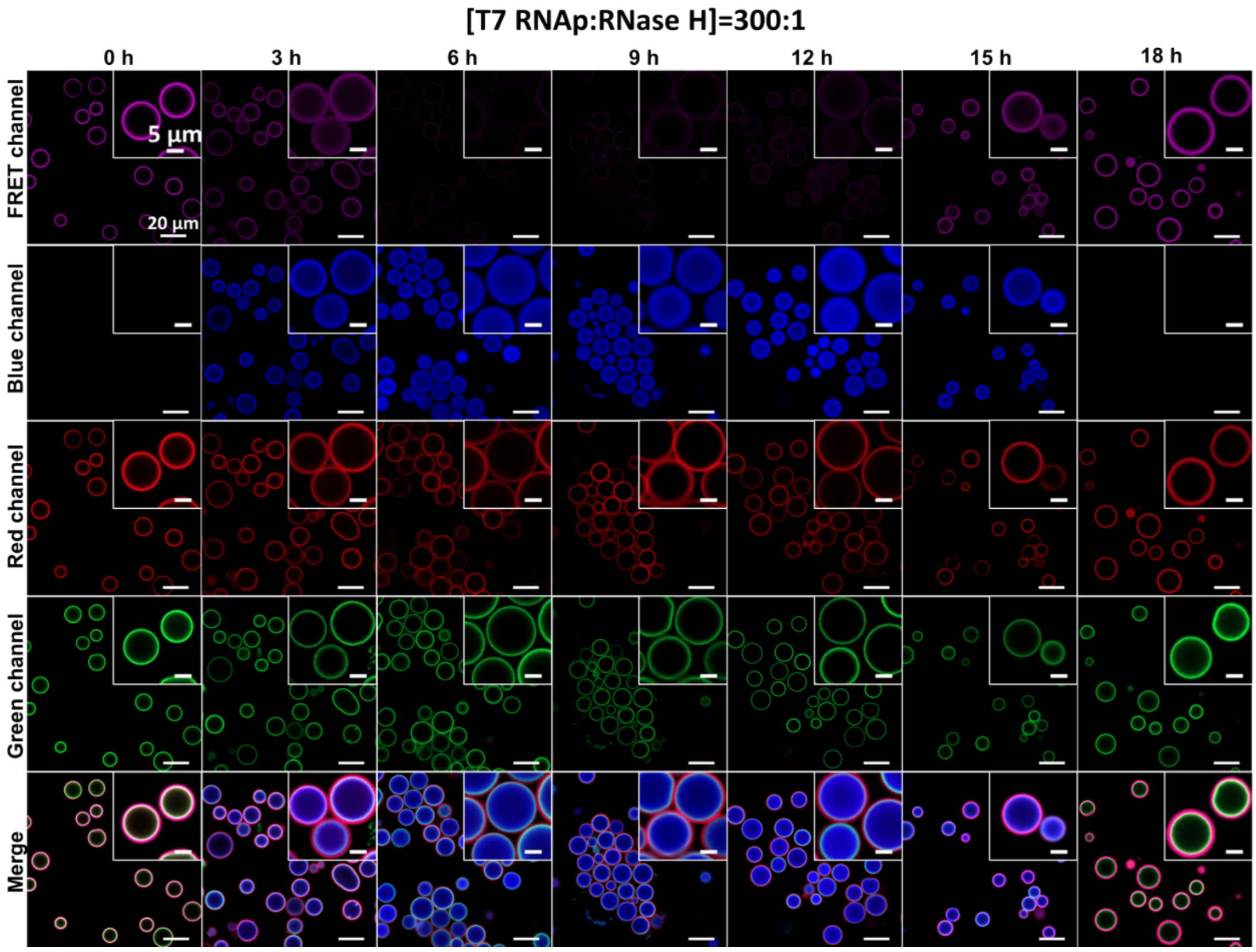

Figure S19. Confocal fluorescence microscopy images corresponding to the transient, dissipative, and temporal reconfiguration of microcompartmentalized MDs, accompanied by aggregation/deaggregation, from state O to state P, and back to state O, driven by T7 RNAp/RNase H, at a T7 RNAp concentration of 2 U/μL and RNase H concentration of 0.0067 U/μL. The derived temporal integrated violet FRET signal changes, and blue fluorescence changes of the compartmentalized MDs correspond to Figure 6(C), curve (ii) and curve (v), respectively. The derived temporal average aggregation counts of the MDs corresponds to Figure 6(D), curve (b) (scale bar = 20 μm, inset scale bar = 5 μm).

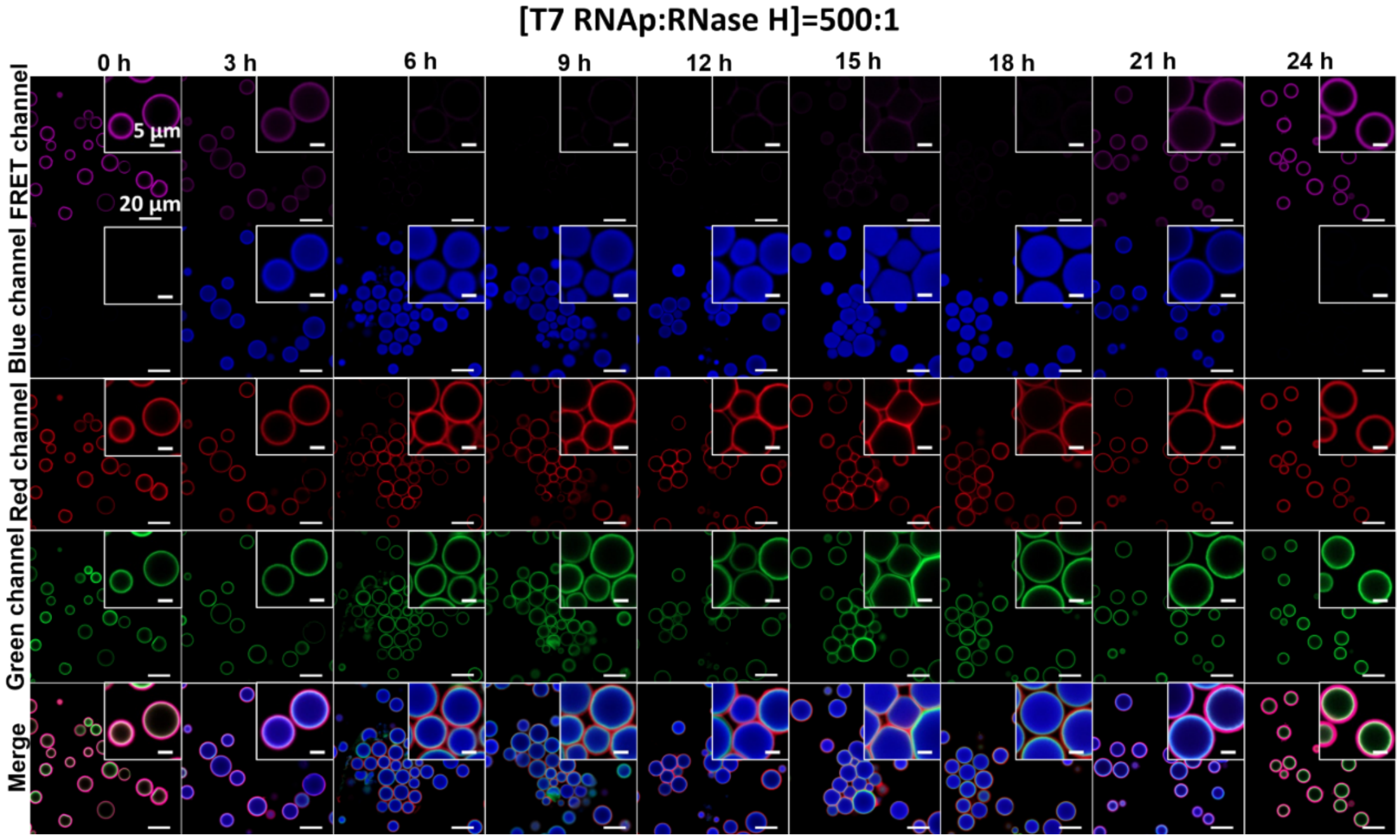

Figure S20. Confocal fluorescence microscopy images corresponding to the transient, dissipative, and temporal reconfiguration of microcompartmentalized MDs, accompanied by aggregation/deaggregation, from state O to state P, and back to state O, driven by T7 RNAp/RNase H, at a T7 RNAp concentration of 2 U/μL and RNase H concentration of 0.004 U/μL. The derived temporal integrated violet FRET signal changes, and blue fluorescence changes of the compartmentalized MDs correspond to Figure 6(C), curve (iii) and curve (vi), respectively. The derived temporal average aggregation counts of the MDs corresponds to Figure 6(D), curve (c) (scale bar = 20 μm, inset scale bar = 5 μm).

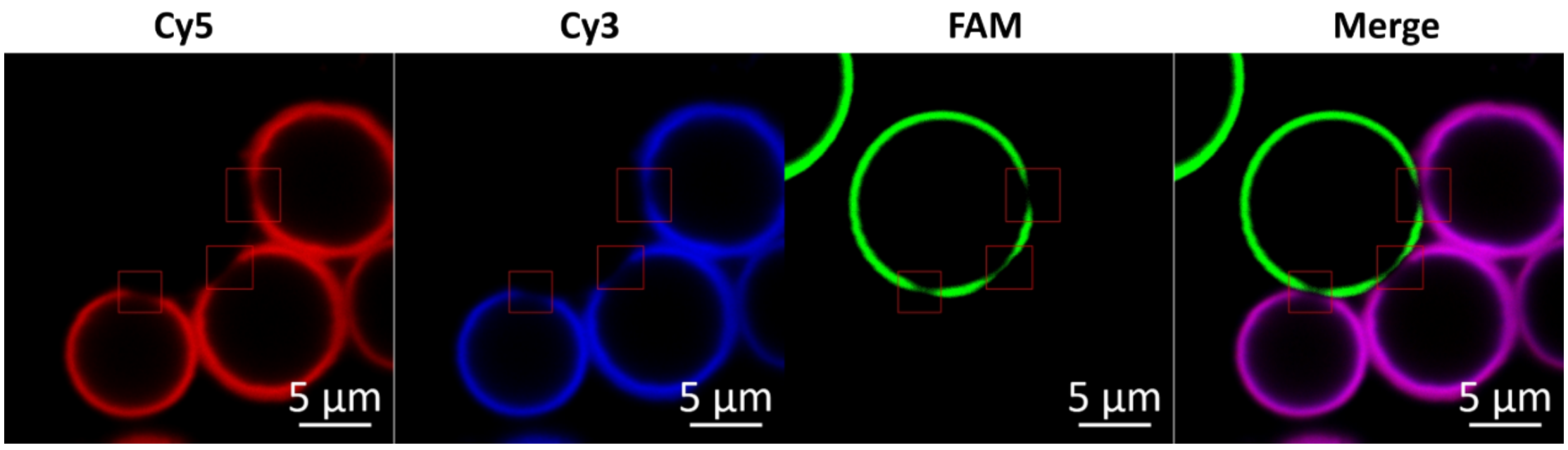

Figure S21. The high-resolution confocal fluorescence microscopy images corresponding to the aggregated condensates W/V in state Z, upon light-triggered activation of the transcription machinery (scale bar = 5 μm).

To validate the aggregation mechanism of MDs W/V by complementary Bs1/Bs1' hybridization, instead of physical contacting, the high-resolution fine structure of the aggregated MDs W/V, upon light-triggered activation of the transcription machinery transferring intercommunicating RNA strand R4 from W to V, was analyzed. Obviously, the fluorescence of Cy3 and Cy5 associated with the compartment T5'-T6'-Bs1 in MDs W showed weaker intensities at the boundaries that contacts the adjacent MDs V. The locally weaker fluorescence is due to the occupation of the barcode Bs1 by adjacent V forming the Bs1/Bs1' crosslinking bridging units. Similarly, the locally weaker fluorescence of FAM associated with the R4-S5'-Bs1' compartment in MDs V at the boundaries that contacts the adjacent MDs W, is also observed, due to the occupation of the Bs1' by Bs1/Bs1' bridging units. The experiments support the aggregated condensates W/V through the formation of complementary barcode Bs1/Bs1' duplex bridging units.

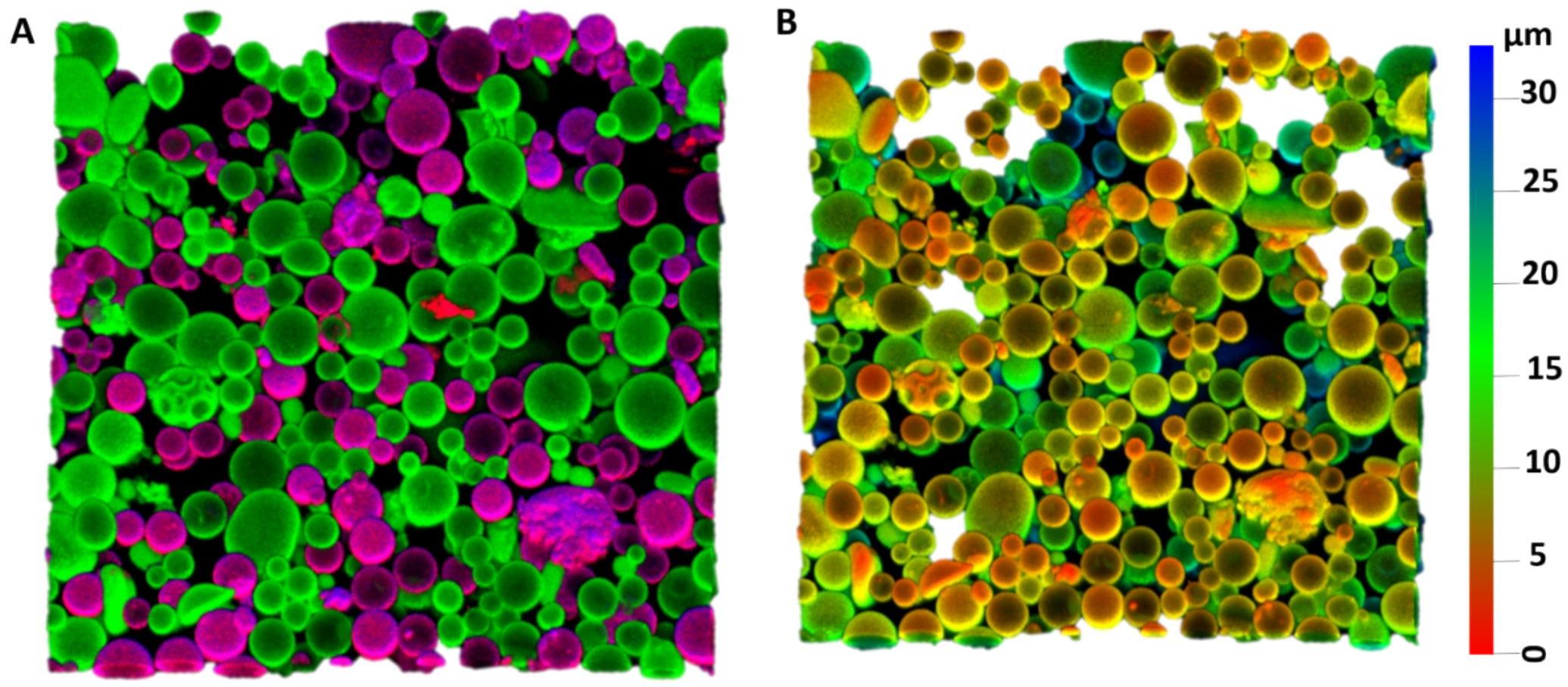

Figure S22. (A) The 3D-stack confocal fluorescence microscopy image of aggregated MDs W/V, upon the light and transcription machinery reconfiguration, and (B) the corresponding depth distribution maps.

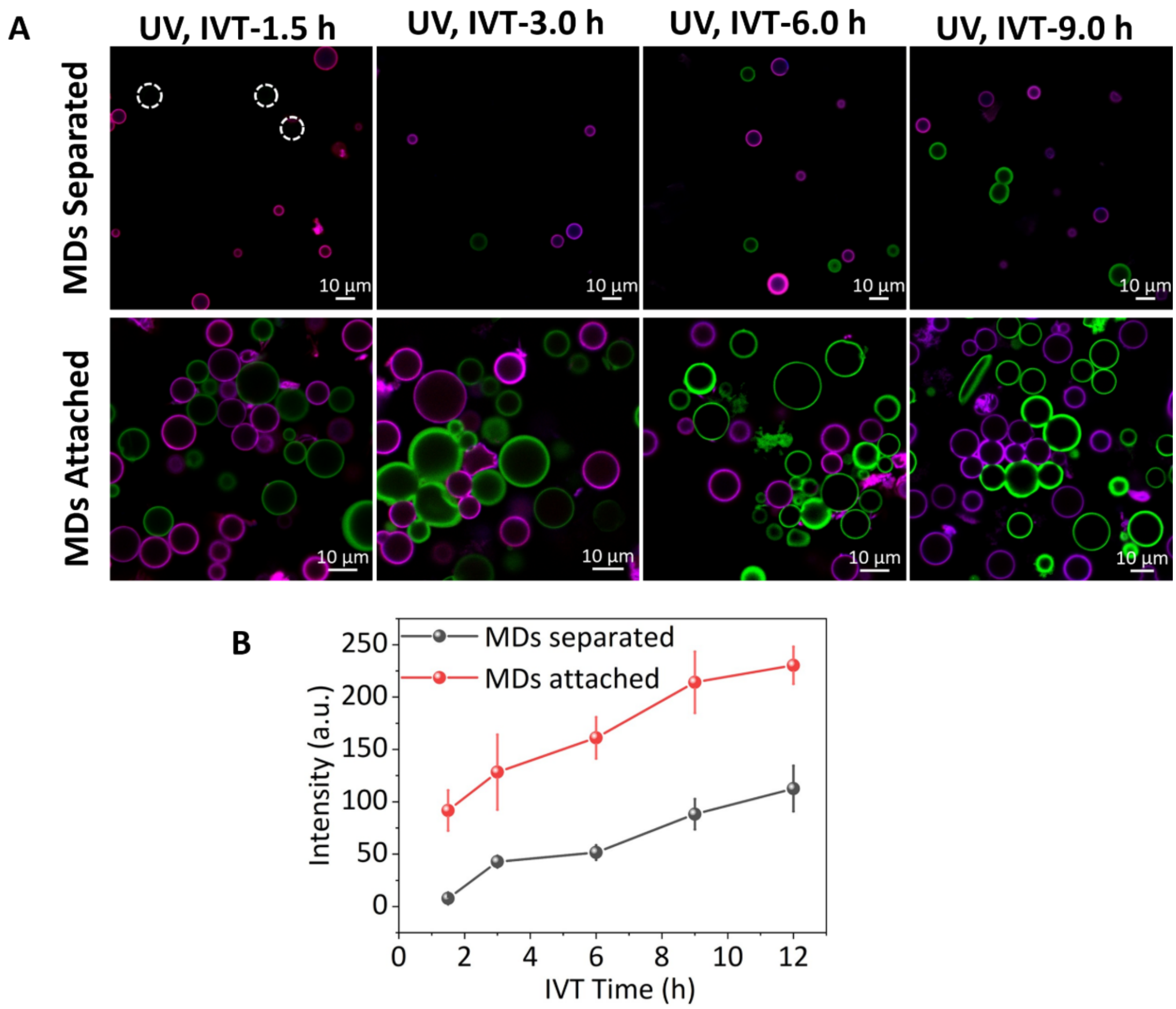

Figure S23. (A) Temporal confocal fluorescence microscopy images of the aggregated MDs W/V, and the control system of separated MDs W'/V', upon the light-triggered, transcription machinery-guided activation of the intercommunicating RNA strand translocated from W to V in aggregated MDs, or W' to V' in aggregated MDs (scale bar = 10 μm). (B) Temporal integrated green fluorescence intensities corresponding to the compartmentalized MDs V or V'. (Error bars extracted from N = 3 experiments for each experiment analyzing 5 large-area 135×135 μm frames total 56 condensates)

To justify the facilitated intercommunicating in aggregated condensates W/V by transcription machinery transforming MDs in state Y to state Z, a control system was also constructed, where separated condensates W'/V' were reconfigured by the transcription

machinery translocating RNA strand from W' to V'. The separated condensates W' and V' were prepared by invading Bs1-modified condensate by T1 and T2 strands, and invading Bs1'-modified condensates by S5 strands, respectively, followed by the mixing of the two compartmentalized condensates W' and V'. As the exterior barcode Bs1 associated with MDs W' was occupied by invading strand T2 and exterior barcode Bs1' associated with the MDs V' was occupied by invading strand S5, the mixing of the MDs W' and V' would not generate available Bs1/Bs1' bridging units that induce the aggregation of the two MDs, leading to the control system of separated compartmentalized MDs W' and V'. As shown in Figure S21(A), the temporal confocal fluorescence microscopy images correspond to the aggregated condensates W/V, or control system, separated condensates W'/V', reconfigured by intercommunicating RNA strand, upon activation of the transcription machinery. The corresponding temporal integrated green fluorescence intensity changes corresponding to R4-S5'-Bs1' constituents in condensates V or constituent R4-S5'-Bs1' in condensates V' were quantitatively evaluated in Figure S23(B). Obviously, the green fluorescence intensity changes of the R4-S5'-Bs1' constituents in condensates V are much more significant than the control system, in condensates V', due to the facilitated transfer of intercommunicating RNA strand in aggregated condensates W/V, in compassion to the separated condensates W'/V'.

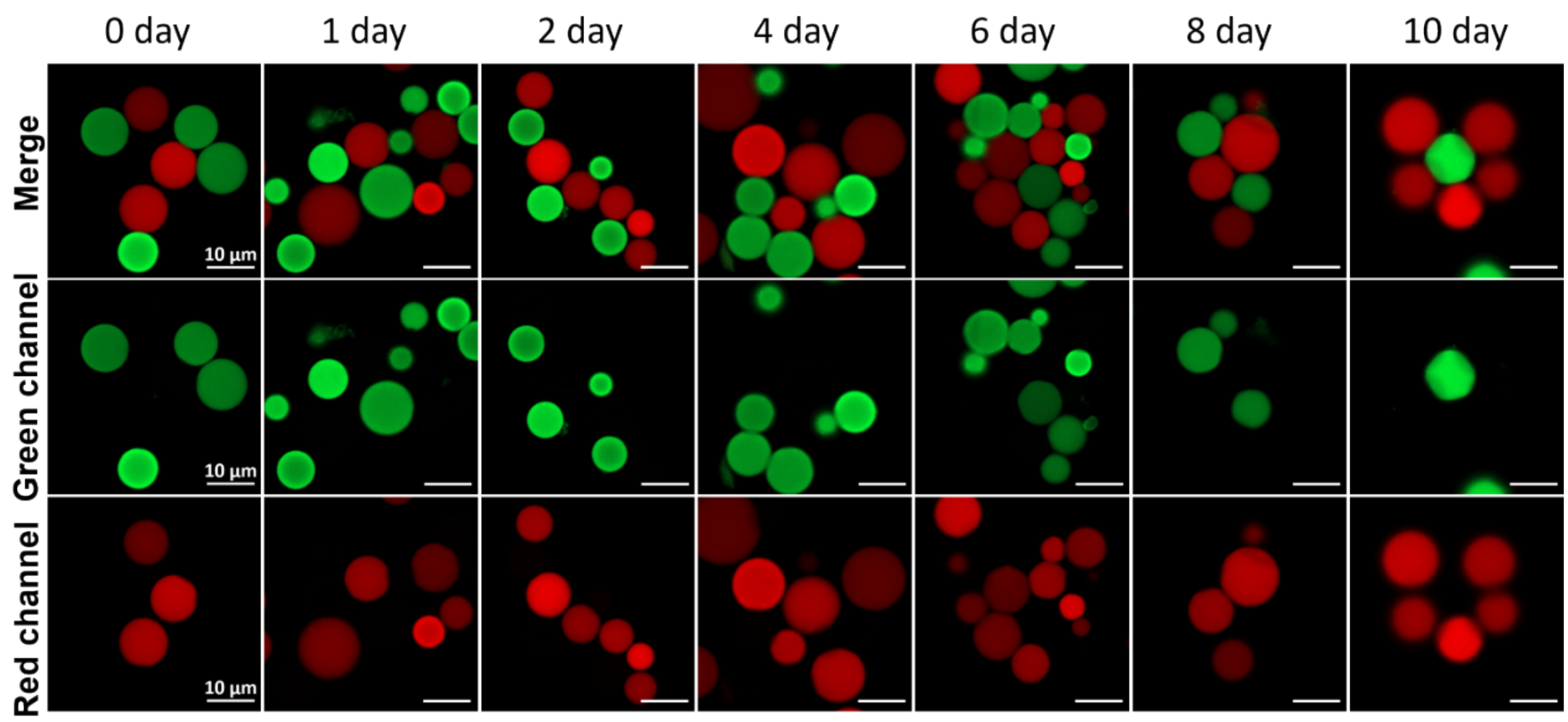

Figure S24. Time-dependent confocal fluorescence images of aggregated MDs, composing of condensate W and condensate V (Figure 7) correspondingly invaded by FAM labeled strand C and Cy5 labeled strand D.

References:

1. M. Delarue, G. P. Brittingham, S. Pfeffer, I. V. Surovtsev, S. Pinglay, K. J. Kennedy, M. Schaffer, J. I. Gutierrez, D. Sang, G. Poterewicz, J. K. Chung, J. M. Plitzko, J. T. Groves, C. Jacobs-Wagner, B. D. Engel and L. J. Holt, *Cell*, 2018, **174**, 338-349.e320.
2. E. Sokolova, E. Spruijt, M. M. K. Hansen, E. Dubuc, J. Groen, V. Chokkalingam, A. Piruska, H. A. Heus and W. T. S. Huck, 2013, **110**, 11692-11697.
3. K. Watanabe, K. Morishita, X. Zhou, S. Shiizaki, Y. Uchiyama, M. Koike, I. Naguro and H. Ichijo, *Nat. Commun.*, 2021, **12**, 1353.
4. P. E. A. Ash, S. Lei, J. Shattuck, S. Boudeau, Y. Carlomagno, M. Medalla, B. L. Mashimo, G. Socorro, L. F. A. Al-Mohanna, L. Jiang, M. M. Öztürk, M. Knobel, P. Ivanov, L. Petrucelli, S. Wegmann, N. M. Kanaan and B. Wolozin, 2021, **118**, e2014188118.
5. N. Avakyan, A. A. Greschner, F. Aldaye, C. J. Serpell, V. Toader, A. Petitjean and H. F. Sleiman, *Nat. Chem.*, 2016, **8**, 368-376.
